# Dendrimeric nanosystem consistently circumvents heterogeneous drug response and resistance in pancreatic cancer

**DOI:** 10.1002/EXP.20210003

**Published:** 2021-08-27

**Authors:** Juan Liu, Chao Chen, Tuo Wei, Odile Gayet, Céline Loncle, Laurence Borge, Nelson Dusetti, Xiaowei Ma, Domenico Marson, Erik Laurini, Sabrina Pricl, Zhongwei Gu, Juan Iovanna, Ling Peng, Xing‐Jie Liang

**Affiliations:** ^1^ CNRS, Centre Interdisciplinaire de Nanoscience de Marseille, UMR 7325, Equipe Labellisée Ligue Contre le Cancer Aix‐Marseille Université Marseille France; ^2^ Laboratory of Controllable Nanopharmaceuticals Chinese Academy of Sciences (CAS) Center for Excellence in Nanoscience Beijing China; ^3^ CAS Key Laboratory for Biomedical Effects of Nanomaterials and Nanosafety National Center for Nanoscience and Technology of China Beijing China; ^4^ University of Chinese Academy of Sciences Beijing China; ^5^ Hepato‐Pancreato‐Biliary Center, Beijing Tsinghua Changgung Hospital, School of Medicine Tsinghua University Beijing China; ^6^ Centre de Recherche en Cancérologie de Marseille, INSERM, UMR1068 Marseille France; ^7^ Molecular Biology and Nanotechnology Laboratory (MolBNL@UniTS), DEA University of Trieste Trieste Italy; ^8^ Department of General Biophysics, Faculty of Biology and Environmental Protection University of Lodz Lodz Poland; ^9^ National Engineering Research Center for Biomaterials Sichuan University Chengdu China

**Keywords:** dendrimeric nanosystem, drug resistance, fluorinated amphiphilic dendrimer, micelle nanostructure, pancreatic ductal adenocarcinoma

## Abstract

Pancreatic ductal adenocarcinoma (PDAC) is a deadly cancer with no efficacious treatment. The application of nanomedicine is expected to bring new hope to PDAC treatment. In this study, we report a novel supramolecular dendrimeric nanosystem carrying the anticancer drug doxorubicin, which demonstrated potent anticancer activity, markedly overcoming the heterogeneity of drug response and resistance of primary cultured tumor cells derived from PDAC patients. This dendrimer nanodrug was constructed with a fluorinated amphiphilic dendrimer, which self‐assembled into micelles nanostructure and encapsulated doxorubicin with high loading. Because of the fine nanosize, stable formulation and acid‐promoted drug release, this dendrimeric nanosystem effectively accumulated in tumor, with deep penetration in tumor tissue and rapid drug uptake/release profile in cells, ultimately resulting in potent anticancer activity and complete suppression of tumor growth in patient‐derived xenografts. Most importantly, this dendrimer nanodrug generated uniform and effective response when treating 35 primary pancreatic cancer cell lines issued from patient samples as a robust platform for preclinical drug efficacy testing. In addition, this dendrimer nanodrug formulation was devoid of adverse effects and showed excellent tolerability. Given all these uniquely advantageous features, this simple and convenient dendrimer nanodrug holds great promise as a potential candidate to treat the deadly PDAC.

## Graphical abstract

A novel supramolecular dendrimeric nanosystem carrying the anticancer drug doxorubicin was developed in this study. We demonstrated this nanosystem markedly overcoming the heterogeneity of drug response and resistance of primary cultured tumor cells derived from 35 PDAC patients. This simple and convenient dendrimer nanodrug holds great promise as a potential candidate to treat the deadly PDAC.

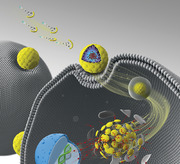

## 1 INTRODUCTION

Pancreatic cancer, in particular pancreatic ductal adenocarcinoma (PDAC), is currently one of the most lethal and devastating human malignancies. Despite decades of efforts, little improvement has been achieved: the overall survival rate is less than 10% and the median survival time is less than 6 months.^[^ ^1^, ^2^, ^3^ ^]^ As most PDACs are typically diagnosed at advanced stages, many PDAC cases (>80%) are not eligible for surgery. Therefore, chemotherapy is often the only therapeutic strategy. The current first‐line PDAC therapeutics includes gemcitabine alone or gemcitabine combined with nab‐paclitaxel (Gem/Nab‐PTX), and the FOLFIRINOX treatment, composed of 5‐fluorouracil/leucovorin, oxaliplatin, and irinotecan.^[^ ^4^, ^5^ ^]^ Also, PDAC is a disease with considerable molecular heterogeneity, which results in diverse clinical outcomes and responses to therapy:^[^ ^6^, ^7^, ^8^ ^]^ nearly 70% and 90% of patients do not respond to FOLFIRINOX and gemcitabine, respectively. In addition, the impenetrable stroma and hypovascularized tumor microenvironment limit drug delivery into PDAC cells, further reducing the treatment effectiveness. Importantly, PDAC is predicted to move to second place in terms of cancer death by 2030.^[^ ^9^ ^]^ Therefore, there is a dire need to design more effective therapeutic strategies for improving treatment efficacy and patient responsiveness.

Therapeutics based on the application of nanotechnology, also known as nanomedicine, are expected to fulfill the promise of bringing new hope and major breakthroughs in cancer treatment, and especially for the hardly manageable PDAC.^[^ ^10^, ^11^ ^]^ This is mainly related to the ability of nanotherapeutics to penetrate and accumulate within tumor lesions via the so‐called enhanced permeability and retention (EPR) effect. The EPR effect originates from the defective lymphatic drainage and leaky vasculature, which are characteristics of tumor microenvironments.^[^ ^12^, ^13^, ^14^ ^]^ Exploiting the EPR effect by nanomedicines with physicochemical characteristics (including optimal charge, size, shape, and drug loading/release) aptly tailored to protect the anticancer chemotherapeutics from premature degradation and release, and to traverse various biological barriers to boost drug delivery specifically in cancer cells within the tumor lesions, can ultimately lead to increased drug efficacy, circumventing heterogeneous drug responses and resistance, as well as reducing toxicity and side‐effects.^[^ ^15^, ^16^, ^17^, ^18^, ^19^, ^20^ ^]^ Therefore, various nanodrug formulations have been developed and explored for treating PDAC. In 2013, the nanoformulation of albumin‐bound paclitaxel (nab‐paclitaxel) combined with gemcitabine has been approved by FDA as the frontline treatment for PDAC. Another nanomedicine, MM‐398, was approved in 2015 for use with folinic acid and 5‐fluorouracil as the second‐line therapeutic plan of metastatic PDAC. Although both combination therapies showed modest improvement in patient overall survival, the corresponding monotherapies did not result statistically significant improvement compared with the standard treatments. Therefore, new nanoformulations of chemotherapeutics with high potency are in high demand.

We have recently reported an innovative and highly effective supramolecular nanomicellar drug formulation based on the spontaneous self‐assembly of small amphiphilic dendrimers.^[^ ^21^ ^]^ These dendrimer nanomicelles harness both the advantages offered by the self‐organizing ability of lipid and the peculiar structure and stability of dendrimer for high drug loading capacity (>40%) yet preserving small size and stable formulation. Although this nanodrug exhibited excellent anticancer performance, its drug release profile remained suboptimal. Here we report a novel dendrimer nanomicellar formulation of the anticancer drug doxorubicin (Dox), which demonstrated potent anticancer activity against PDAC. Specifically, we have designed and identified a novel amphiphilic dendrimer bearing a fluorinated entity on the hydrophobic portion. This fluorinated amphiphilic dendrimer (FAD) was able to self‐assembly into spherical nanomicelles to encapsulate the cytotoxic anticancer drug Dox, with excellent loading efficiency. The resulting nanomicellar system presented remarkable features such as: (i) excellent stability to prevent premature drug release along the delivery pathway to cancer cells under physiological condition, (ii) small size, resulting in effective accumulation and deep penetration into tumor lesions via EPR effect, and (iii) efficient drug release in the acidic tumor microenvironment to boost local drug concentration. Most importantly, this nanodrug system successfully circumvented heterogeneous drug response/resistance in our proof‐of‐concept study performed using primary human pancreatic cancer cells derived from 35 different patient tumor samples. These primary cancer cells typically present heterogeneous responses to clinical anticancer drugs;^[^ ^22^ ^]^ as such, they represent reliable models for assessing anticancer activity and efficacy closely reflecting the real situation of pancreatic cancer. Remarkably, when compared to free Dox, our Dox‐loaded nanomicelles demonstrated an effective and consistent anticancer effect on all the 35 primary cancer cells tested. Most importantly, they were able to significantly suppress tumor growth in mice with xenografts derived from patient tumors (PDXs) while being devoid of adverse effects. Altogether, these results provide evidence that our FAD/Dox nanodrug constitutes an effective and promising translational candidate to fight against PDAC.

## 2 MATERIALS AND METHODS

### 2.1 Preparation of nanomicelles

AD‐Dox and FAD‐Dox loaded nanomicelles were prepared using amphiphilic dendrimer and Dox by the film dispersion method. The drug, Dox, was dissolved in 1 mL mixed solvent (chloroform:methanol = 3:2, vol/vol, molar ratio of Dox:triethylamine = 1:3) and mixed with amphiphilic dendrimer in 3 mL mixed solvent (chloroform:methanol = 3:2, vol/vol). Their mixed ratio is shown in Table S1. The solvent was removed by vacuum rotary evaporation to form a dry drug‐containing lipid film. And then, this film was hydrated with phosphate buffered saline (PBS) for 30 min at 60°C. Non‐encapsulated Dox was separated by filtration of the micelle suspension through a 0.22 µm polycarbonate membrane (Merck Millipore). The empty nanomicelles were prepared in an identical procedure except that no drug was present in the mix solvent. For fluorescent in vivo imaging, the near‐infrared fluorescent probe, DiR, was encapsulated into nanomicelles. The DiR‐loaded nanomicelles were prepared using the same procedures as Dox loading.

### 2.2 Characterization of nanomicelles

#### 2.2.1 Drug encapsulation efficiency and loading content

Dox concentration in micelles was determined using a spectrometer with Ex = 485 nm and Em = 592 nm. The drug encapsulation efficiency and drug‐loading content were calculated as reported below: encapsulation efficiency (%) = Wt/Wi × 100%, drug loading content (%) = Wt/Ws × 100%, in which Wt represents the amount of Dox loaded into nanomicelles, Wi is the initial amount of Dox fed, and Ws represents the amount of drug loaded nanomicelles after lyophilization.

#### 2.2.2 Micelle morphology characterization

The morphology of nanomicelles was determined using transmission electron microscope (TEM). Briefly, 5 µL of a solution of AD‐Dox, FAD‐Dox, AD, or FAD were dropped on the carbon grid, and then dried for 1 h. The grid was subsequent stained with uranyl acetate. Imaging was performed using a FEI Tecnai G2 20 TWIN microscope. Data were analyzed with the Digital Micrograph software.

#### 2.2.3 Micellar size characterization

Hydrodynamic sizes of the nanomicelles were measured in aqueous solution by dynamic light scattering (DLS) (Zetasizer 5000, Malvern).

#### 2.2.4 Interaction of dox with AD and FAD micelles

The interaction of Dox with FAD and AD micelles was also characterized by ITC experiments in PBS at pH 5 and pH 7.4, respectively. In this case, 19 injections were applied. The AD or FAD concentration in the calorimetric cell was 50 µm, while the Dox concentration in the syringe was equal to 4 mm.

### 2.3 In vitro drug release

Release of Dox from AD‐Dox or FAD‐Dox was determined by the dialysis method. 200 µL of AD‐Dox or FAD‐Dox (1 mg/mL) were dialyzed at 37°C in PBS buffer of pH 7.4 and pH 5.0. Dox concentration was measured using a LS 55 luminescence spectrometer (Perkin‐Elmer). Accumulative release of Dox from AD‐Dox or FAD‐Dox nanomicelles was expressed as percentage of released Dox and plotted as a function of time.

### 2.4 In vitro antiproliferation activity assay

The antiproliferation activity of free Dox, the clinical nanodrug Caelyx, and the AD‐Dox, FAD‐Dox, AD or FAD nanomicelles against human PDAC cancer cells was evaluated using the Alamar blue assay (Cell Viability Reagent, Invitrogen). Cells were seeded at 5 × 10^3^ cells per well in a 96‐well plate, preincubated for 24 h, then incubated with the different Dox formulation for 24 h at drug concentrations ranging from 0.01 to 100 µg/mL. The medium was added directly with 10 µL Alamar blue reagent and after 2 h of incubation at 37°C, the fluorescent intensity of samples was measured using an Infinite M200 microplate reader (Tecan) at wavelengths of 530 nm for excitation and 590 nm for emission. Untreated cells were used as control.

### 2.5 Internalization of free dox and FAD‐Dox nanomicelles

Internalization of free Dox and FAD‐Dox nanomicelles in PDAC087T and PDAC074T cells was examined using confocal microscopy and flow cytometry.

For confocal microscopy, 1.5 × 10^4^ cells per well were seeded into confocal dishes and incubated at 37°C overnight. Next, the medium was removed and replaced with solutions of free Dox and FAD‐Dox nanomicelles, respectively, at a final Dox concentration of 10 µg/mL at 37°C. After 1 or 4 h incubation, cells were fixed using 4% wt/vol formaldehyde solution for 15 min, followed by DAPI staining to the nuclei, and then imaging using a confocal microscope (LSM 510 META, Leica).

For flow cytometry experiments, 1.5 × 10^5^ cells per well were seeded into six‐well plates and incubated at 37°C overnight. The medium was then removed and replaced with free Dox and FAD‐Dox nanomicelles at a final concentration of 10 µg/mL for 5, 10, 20, 40, 60, 120, and 240 min at 37°C. Finally, the cells were harvested and washed with 1× PBS solution three times and then analyzed by flow cytometry (MACS, Miltenyi Biotec). Each assay was performed in triplicate.

### 2.6 Endocytosis inhibition experiments

PDAC087T and PDAC074T cells were seeded at a density of 1.5 × 10^5^ cells per well in 6‐well plates. After 24 h incubation in complete medium, cells were incubated with different inhibitors in serum‐free medium for 1 h. Next, the medium was replaced with complete medium containing free Dox and FAD‐Dox nanomicelles, respectively, at drug concentrations of 10 µg/mL and the same corresponding inhibitor for another hour. The control group was incubated in serum‐free medium without inhibitors before the addition of free Dox and FAD‐Dox nanomicelles. After treatment, all the samples were collected and tested by flow cytometry.

To evaluate the energy dependent pathway, the cells were pretreated in serum‐free medium at 4°C for 1 h. After that, cells were incubated in complete medium containing free Dox and FAD‐Dox nanomicelles at 4°C for 1 h. The different cell‐uptake inhibitors were added at the following final concentrations: chlorpromazine hydrochloride 10 µg/mL (an endocytotic inhibitor of clathrin‐mediated endocytosis), cytochalasin D 10 mm (an endocytotic inhibitor of macro macropinocytosis‐mediated endocytosis), and genistein 10 mm (an endocytotic inhibitor of caveolae‐mediated endocytosis).

### 2.7 Subcellular localization of nanomicelles

PDAC087T and PDAC074T cells were seeded into 35 mm glass dishes, incubated at 37°C for 24 h, then cultured with free Dox and FAD‐Dox nanomicelles at Dox concentration of 10 µg/mL for 10 min, 30 min, 1 h, 2 h, and 4 h at 37°C. Cells were imaged using confocal microscopy. Lysotracker Green DND‐26 was used to stain endosomes and lysosomes. Hoechst 33342 was used to stain the nucleus.

### 2.8 In vivo anticancer activity assay

For tumor inhibition activity in vivo studies, at day 0 NMRI mice were injected subcutaneously in the right flank with a PDAC087T or PDAC074T cell suspension containing 1 × 10^7^ cells with Matrigel. After 10 days, the mice were randomized into the following six treatment groups: physiological saline, FAD, free Dox (2.5 mg/kg), free Dox (5.0 mg/kg), FAD‐Dox (2.5 mg/kg), and FAD‐Dox (5.0 mg/kg). The FAD and FAD‐Dox groups were administered at a Dox equivalent dose. At regular day intervals, these samples were administered intravenously to the mice. Tumor progression in the mice was monitored, and tumor volumes were calculated using the following formula: Volume = 1/2 × *LW* ^2^, in which *L* and *W* are the longest and shortest tumor diameters, respectively.

After mice were sacrificed, all organs were extracted and paraffin sections were prepared from the tumors. The paraffin sections of all tumors were stained by anti–Ki‐67 monoclonal antibody to evaluate tumor proliferation, and anti‐caspase three monoclonal antibody to assess cell apoptosis. The paraffin sections of all organs were performed with H&E staining for histological analysis.

### 2.9 In vivo biodistribution

FAD‐DiR nanomicelles were prepared using the same method described above. To determine their in vivo biodistribution, female NMRI mice bearing PDAC087T tumors were injected intravenous (i.v.) with 100 µL of PBS, free DiR, and FAD‐DiR nanomicelles at a dose corresponding to 8.0 µg/mL of DiR via tail vein (*n* = 4 per group). The real‐time distribution and tumor accumulation of PBS, free DiR, and FAD‐DiR nanomicelles were recorded at 0.5, 2, 4, 8, 12, 24, 36, and 48 h post injection using an in vivo imaging system (Photon Imager; Biospace Lab). After 48 h, mice were sacrificed and the organs were removed for ex vivo fluorescence imaging.

The isolated tumors were embedded in optimal cutting temperature compound, and cut into 6 µm slices. Tumor vessels in frozen sections were recognized by the expression of CD31 on endothelial cells. Sections were fixed in 4% paraformaldehyde for 20 min, washed three times in PBS, blocked with 10% BSA for 30 min and then incubated with rat anti‐CD31 (Abcam) antibody overnight at 4°C. Sections were washed in PBS and stained with a FITC‐conjugated goat anti‐rat IgG secondary antibody for 1 h. Sections were again washed, covered with coverslip, and observed using a confocal microscope.

### 2.10 Pharmacokinetics study

For pharmacokinetics studies, free Dox (10 mg/kg) or FAD‐Dox nanomicelles (10 mg/kg, Dox equivalents) was i.v. injected into NMRI healthy mice (*n* = 6 for each group). Blood was collected at regular times after injection from the mice tail vein and the plasma was separated and analyzed for Dox concentration. The 10 µL plasma samples were incubated with 490 µL of acidified isopropanol overnight to extract drug. The mixture was then centrifuged at 15,000 g for 20 min. Dox concentrations were measured by HPLC. Dox concentrations were normalized to the protein content as measured by the bicinchoninic acid assay.

### 2.11 Statistical analysis

Data are presented as the mean value ± SD from three independent measurements. For statistical analyses, the Student's *t*‐test was used. Error bars represent standard deviations.

## 3 RESULTS

### 3.1 The fluorinated amphiphilic dendrimer FAD forms stable nanomicelles with the anticancer drug doxorubicin yet promotes effective drug release in the acidic tumor microenvironment

**FIGURE 1:**
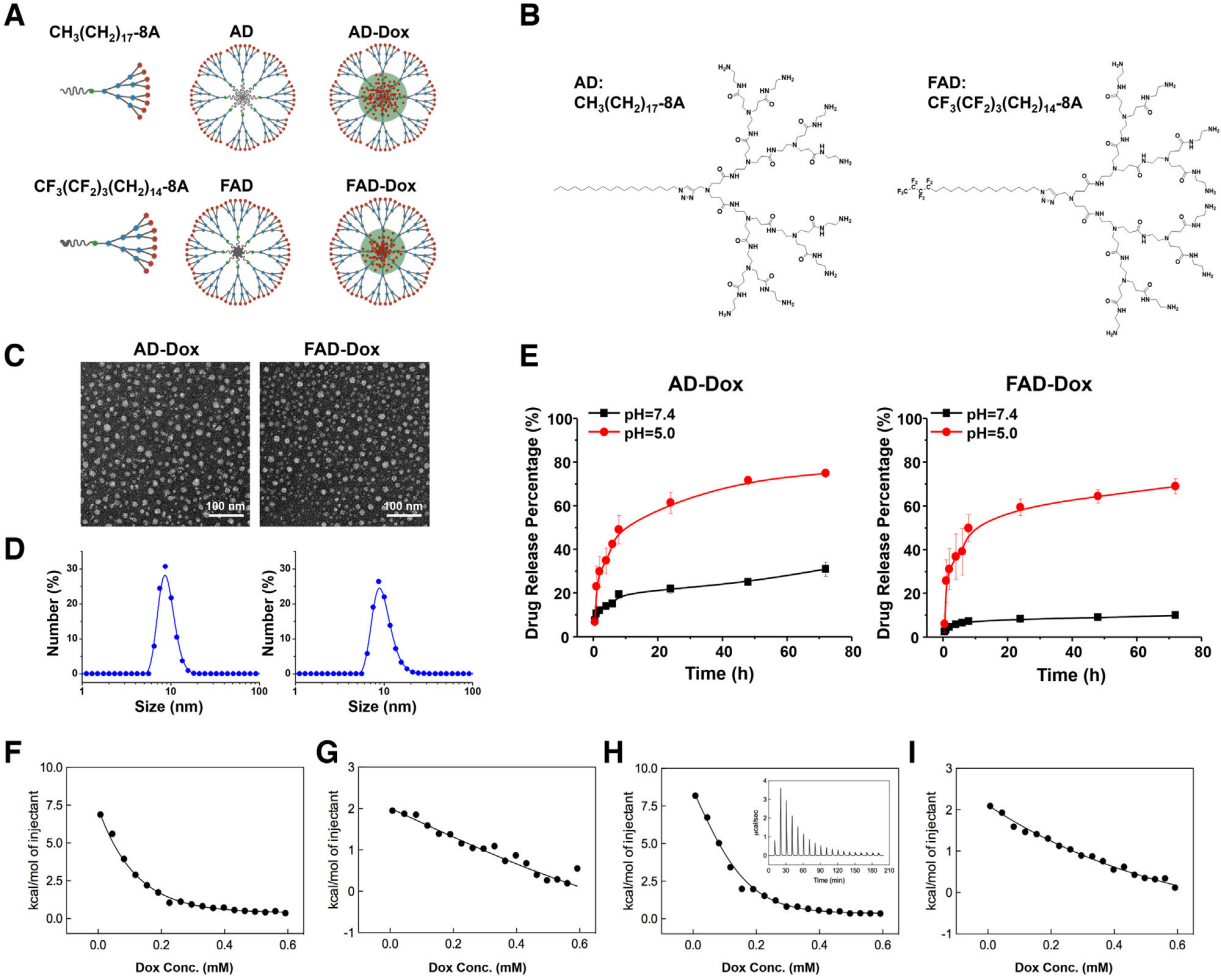
Amphiphilic dendrimers can self‐assemble into nanomicelles to encapsulate the anticancer drug doxorubicin (Dox) and promote its pH‐dependent release. (A) Cartoon illustration of AD and FAD amphiphilic dendrimers self‐assembly into nanomicelles for drug encapsulation. (B) Chemical structure of the amphiphilic dendrimers AD and FAD. (C) TEM images of AD‐Dox and FAD‐Dox nanomicelles. (D) The size distribution of AD‐Dox and FAD‐Dox nanomicelles detected by dynamic light scattering (DLS). (E) Release profiles of Dox from AD‐Dox and FAD‐Dox micelles at pH 7.4 and pH 5.0 at 37°C. (F–I) Representative integrated ITC profiles for the interaction of Dox in PBS buffer with: AD micelles at pH 7.4 (F); AD micelles at pH 5.0 (G); FAD micelles at pH 7.4 (H); and FAD micelles at pH 5.0 (I). The solid lines are data fitting with a sigmoidal function. The inset in panel h shows the corresponding ITC raw data as an example. All experiments were run in triplicate

Our previously established dendrimer nanodrug, although very efficient, was characterized by an unsatisfactory drug release profile.^[^ ^21^ ^]^ In order to construct more effective dendrimer nanosystems against PDAC yet with high drug loading but a better release profile, we studied two amphiphilic dendrimers, normal amphiphilic dendrimer (AD) and FAD. Both dendrimers retain the hydrophilic poly(amido)amine dendron part and one hydrophobic chain, constitute by a C18 alkyl chain for AD, and a fluorinated C18 alkyl chain for FAD, respectively (Figure 1A; Figure S1). These two dendrimers were able to form small nanomicelles (Figure 1B), with the critical micelle concentrations (CMC) of 12.6 µm for AD and 4.4 µm for FAD, respectively (Figure S2). We further characterized AD and FAD micelle formation using isothermal titration calorimetry (ITC) (Figure S3),^[^ ^23^ ^]^ and demonstrated that micelle formation is thermodynamically spontaneous in both cases (Δ*G*_mic_ < 0). In addition, ITC gave CMC values in excellent agreement with those obtained by the fluorescent spectroscopic assay. Specifically, FAD has a CMC (6.01 µm) lower than AD (11.2 µm) (Figure S4). This corresponds to a lower (i.e., more favorable) free energy of micellization Δ*G*_mic_ for FAD (−10.92 kcal/mol) with respect to its non‐fluorinated counterpart AD (−10.33 kcal/mol).

Importantly, AD and FAD had a high loading efficiency for the anticancer drug Dox up to 42% and 36%, respectively (Table S1). The loading efficiency of FAD was a little lower than that of AD, which was consistent with the CMC of them. However, both of them had a high drug encapsulation efficiency (>90%). In addition, both Dox‐loaded dendrimer nanomicelles were small in size, stable, and monodispersed in solution (Figure 1C,D; Figure S5A,B), and they did not exhibit any notable change in size even after storage at 4°C for 6 months in the dark (Figure S5C). All these evidences indicate that both AD‐Dox and FAD‐Dox nanomicelles have excellent drug loading efficiency and stability. In addition, they exhibited efficient drug release at pH 5.0—a condition which mimics tumor acidic microenvironment—with a maximum drug release of 75% at 72 h (Figure 1E). Remarkably, this represents an almost twofold increase in drug release efficiency with respect to our previous dendrimer nanosystem.^[^ ^8^ ^]^ However, for AD‐Dox, the drug release at the physiological pH 7.4 was almost 40% at 72 h, an unfavorable situation for achieving effective and efficient drug delivery exclusively at tumor site while avoiding systemic toxicity related to drug leakage in the circulation during transport. To our great interest, FAD‐Dox micelles showed very low drug release at pH 7.4 (less than 10% at 72 h). This stability of FAD‐Dox nanodrug at neutral environment can be ascribed to unique interactions among the fluorinated hydrophobic components when FAD self‐organize into nanomicelles and encapsulate Dox. The finding that FAD‐Dox nanomicelles are more stable than AD‐Dox nanomicelles at pH 7.4 also nicely correlates with the corresponding CMC values determined by spectroscopic and calorimetric techniques, that is, CMC_FAD_ < CMC_AD_ (Figures S2 and S4).

We further studied the interaction of the two dendrimer nanomicelles with Dox again using ITC in buffered solutions at pH 7.4 or 5.0, respectively (Figure 1F–I; Table S2). Inspection of the thermodynamic parameters listed in Table S3 revealed that, for both systems, Dox/micelle interactions were prevalently entropic in nature. Indeed, while endothermic contributions were measured in all cases, the driving force for Dox binding to either AD or FAD micelles resulted from the strong, positive entropic variations, which ultimately determined the overall negative values of the free energy. More interestingly, however, when considering the ITC data at pH 7.4 we saw that the values of the free energy of interaction Δ*G* (−5.68 kcal/mol) and, consequently, of the dissociation constant between Dox and the FAD micelles *K*_d_ (70 µm) were significantly more favorable than those for the alternative AD‐Dox system (−5.01 and −5.68 kcal/mol, respectively). On the other hand, at pH 5 the strength of the interactions between the drug and both micelle types decreased, as reflected by the plummeted values of the corresponding Δ*G* and *K*_d_ values (Δ*G* = −3.31 and −3.27 kcal/mol, and *K*_d_ = 6700 and 5400 µm for AD‐Dox and FAD‐Dox, respectively). This finding is in line with the corresponding Dox release data at the two pH values. In fact, at pH 7.4 the strongest intermolecular interactions between Dox and the FAD‐based micelles resulted in a substantially slower drug release from this system with respect to that observed for the AD micelles whereas, at the more acidic pH, the interactions between both micelle types and the Dox molecules were almost equally less effective, leading to faster and comparable drug release profiles (Figure 1E).

Based on the overall more advantageous features of the FAD‐Dox nanomicelles, we continued our further studies only on this nanodrug.

### 3.2 FAD‐dox nanomicelles exhibit effective and consistent anticancer activity on 35 primary human pancreatic cancer cells via rapid and efficient macropinocytosis‐mediated cellular uptake

**FIGURE 2:**
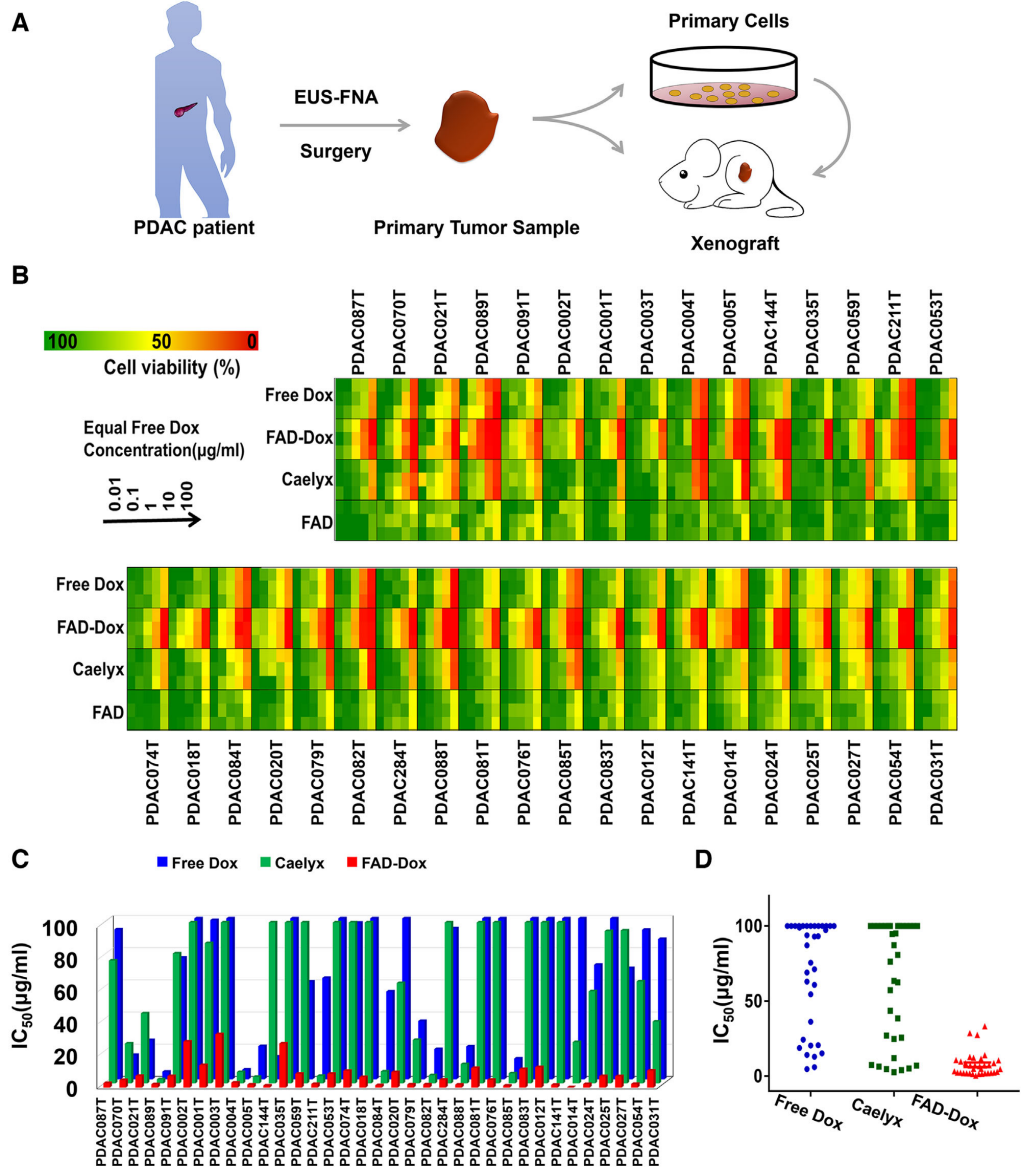
FAD‐Dox nanomicelles show effective and consistent antiproliferative activity on primary human pancreatic cancer cell lines derived from patients. (A) Scheme showing the process of obtaining human PDAC patient‐derived cells and xenografts. EUS‐FNA is endoscopic ultrasound‐guided fine‐needle aspiration. (B) Antiproliferation activity of FAD nanomicelles assessed in 35 primary human pancreatic cancer cell lines in comparison with that of the empty FAD nanomicelles and the clinical anticancer drugs Dox and Caelyx. (C) IC_50_ values and (D) the distribution of IC_50_ values for FAD‐Dox nanomicelles on the 35 primary human pancreatic cancer cells in comparison with those of Dox and Caelyx

We next assessed the anticancer activity of the FAD‐Dox nanomicelles in pancreatic cancer models as a proof‐of‐concept study. As mentioned above, pancreatic cancer is a disease with considerable molecular heterogeneity^[^ ^8^, ^24^, ^25^ ^]^; to account for this tumor characteristics, we used 35 primary human pancreatic cancer derived from patients^[^ ^26^, ^27^ ^]^ to evaluate the anticancer activity of FAD‐Dox (Figure 2A). Among them, 15 cell lines were from patients who survived less than 8 months, and the remaining 20 cell lines were from patients who survived more than 8 months (Figure S6). These primary cells display significant heterogeneity in their responses to common clinical anticancer drugs.^[^ ^28^ ^]^ Remarkably, all the primary cancer cells had a consistent response to our FAD‐Dox nanomicelles, which resulted in effective concentration‐dependent inhibition of cell proliferation (Figure 2B,C). The potency of the FAD‐Dox nanomicelles was considerably higher than that of the clinical drugs free Dox and Caelyx, which is a commercial Dox nanoformulation. The IC_50_ values for Dox and Caelyx ranged from 4.00 µg/mL to more than 100 µg/mL, the average IC_50_ being over 60 µg/mL (Figure 2D). This highlights the considerable heterogeneity of the response and resistance of pancreatic cancer cells to Dox and Caelyx, in line with clinical drug response outcomes. In contrast, the corresponding IC_50_ values of FAD‐Dox were mostly centered below 10 µg/mL, which demonstrates the consistently favorable response of all the 35 primary cancer cells to the FAD‐Dox nanomicelles. These results demonstrate the superior performance of FAD‐Dox compared to the clinical drugs Dox and Caelyx.

**FIGURE 3:**
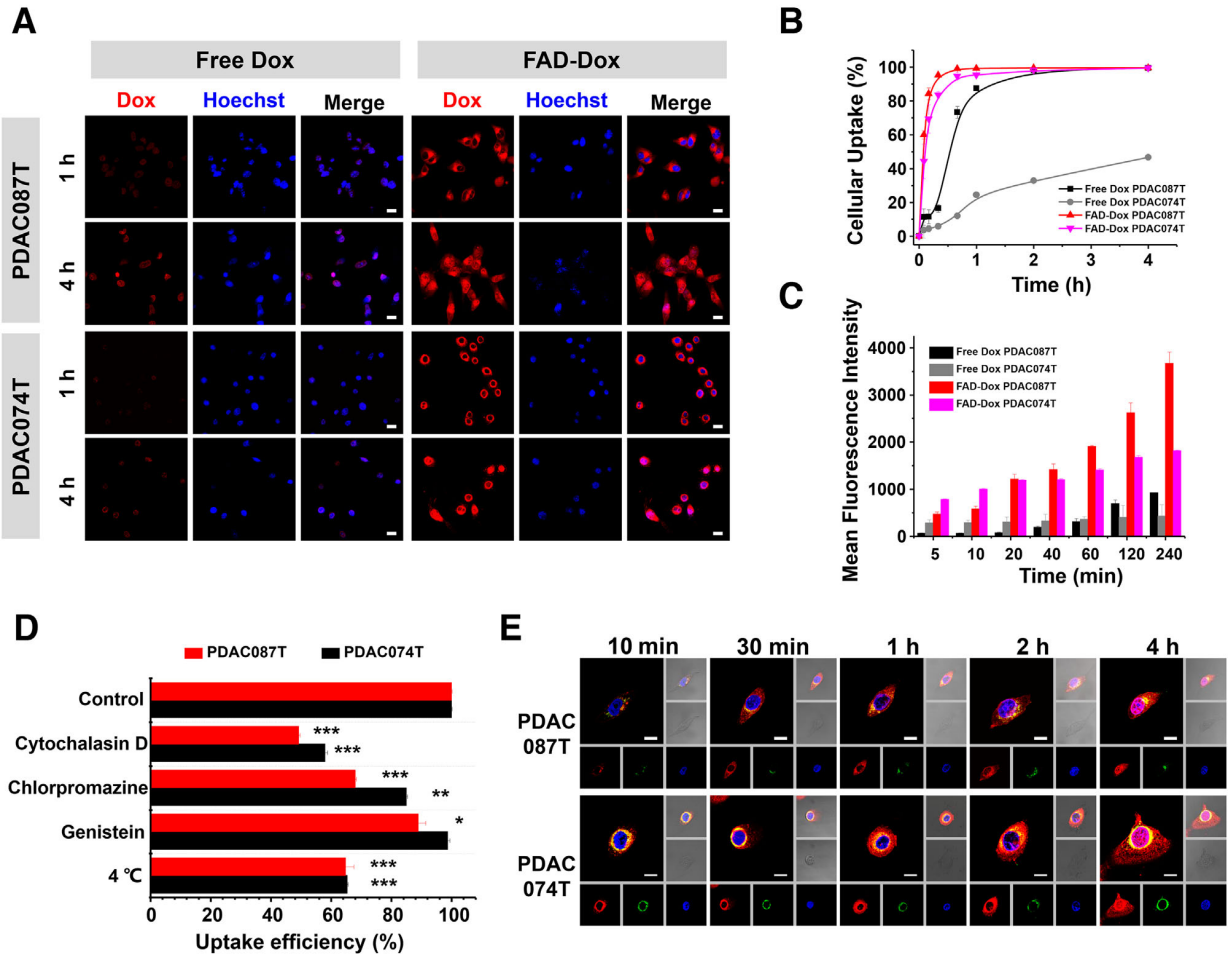
Rapid and effective cell uptake and intracellular traffic of Dox in primary human pancreatic cancer PDAC087T and PDAC074T cells treated with FAD‐Dox nanomicelles. (A) Cellular uptake of free Dox or FAD‐Dox imaged by confocal microscopy following treatment of PDAC087T and PDAC074T cells for 1 and 4 h (scale bar: 10 µm, red: Dox, blue: Hoechst 33342). (B) Kinetics of cellular uptake (%) of free Dox and FAD‐Dox nanomicelles in PDAC087T and PDAC074T cells. (C) Mean fluorescence intensity of Dox in PDAC087T and PDAC074T cells treated with free Dox and FAD‐Dox nanomicelles as function of incubation time. (D) Inhibition of the cell uptake of Dox by PDAC087T and PDAC074T cells treated with FAD‐Dox nanomicelles in the presence of specific endocytosis inhibitors. Cytochalasin D: the inhibitor of macropinocytosis‐dependent endocytosis; Chlorpromazine: the inhibitor of clathrin‐mediated endocytosis; Genistein: the inhibitor of caveolae‐mediated endocytosis. *p < 0.05; **p < 0.01; ***p < 0.001, two‐tailed Student's *t*‐tests. (E) Subcellular localization of Dox was imaged by confocal microscopy following treatment of PDAC087T and PDAC074T cells with free Dox and FAD‐Dox nanomicelles (scale bar: 10 µm, red: Dox, blue: Hoechst 33342, green: lysosome)

In order to get insight into the observed enhanced drug response to the FAD‐Dox nanomicelles, we examined the cellular uptake using PDAC087T and PDAC074T pancreatic cancer cells as representative primary cancer cell models. PDAC087T and PDAC074T cells were derived from patients with short‐ and long‐term survival, respectively. Compared with the clinical drug Dox (left panel in Figure 3A), the FAD‐Dox (right panel in Figure 3A) nanosystem showed much more efficient and rapid uptake by both PDAC087T and PDAC074T cells. This was further confirmed using flow cytometry quantification. Accordingly, the mean fluorescence intensity detected in both these cell lines after 4 h treatment with FAD‐Dox (Figure 3B) was eightfold and fivefold higher, respectively, than that seen after treatment with free Dox (Figure 3C). These results demonstrate that the cellular uptake of FAD‐Dox nanomicelles was significantly faster and more efficient than free Dox; as a consequence, the intracellular Dox concentration was higher in PDAC087T and PDAC074T cells treated with the nanomicelles than in cells treated with the free drug. These data were further confirmed in eight other primary pancreatic cancer cells treated with FAD‐Dox nanomicelles, which showed similar results (Figure S7).

The efficient cellular uptake of FAD‐Dox can be largely attributed to endocytosis, through distinct mechanisms. As displayed in Figure 3D, the fluorescence intensity of cells treated with FAD‐Dox at 4°C was consistently lower than that detected in cells treated at 37°C. This proves that energy‐dependent endocytosis is the major pathway for cell internalization of the FAD‐Dox nanomicelles. Cellular uptake also decreased upon treatment with various endocytosis inhibitors. Cytochalasin D could inhibit the internalization of FAD‐Dox. On the contrary, genistein and chlorpromazine both showed much less inhibition of cellular uptake. All above results indicate that the cellular uptake of FAD‐Dox indeed occurred via a nanoparticle‐based endocytosis pathway, and macropinocytosis, a preferred mechanism for drug delivery, was the predominant pathway for the cellular uptake of the FAD‐Dox nanomicelles. This is also in line with the recent finding that in pancreatic cancer cells and PDAC cells in particular, mutant KRAS‐driven transcriptional reprogramming results in enhanced macropinocytosis to meet the high tumor metabolic needs.^[^ ^28^ ^]^

We next examined the intracellular trafficking of the FAD‐Dox nanomicelles. As shown in Figure 3E, FAD‐Dox rapidly localized in endosomes 10 min post‐incubation. After 30 min, fluorescent Dox was found in both the cytoplasm and endosomes/lysosomes, implying Dox release from the endosomes. Subsequently, Dox translocated into the nucleus at 2 h post‐incubation. Collectively, these data demonstrate that the FAD‐Dox nanomicelles were taken up effectively into cells via macropinocytosis and trafficked through the endosomal/lysosomal system. Dox was then released into the cytoplasm and translocated into the nucleus, where it could intercalate with DNA and consequently exert its cytotoxic effects. The efficient uptake of nanomicelles resulted in a very rapid increase in the intracellular Dox concentration, which markedly inhibited cancer cell proliferation and overcame the heterogeneous drug response and drug resistance.

### 3.3 FAD‐dox dendrimer nanomicelles significantly reduce the toxicity of doxorubicin

**FIGURE 4:**
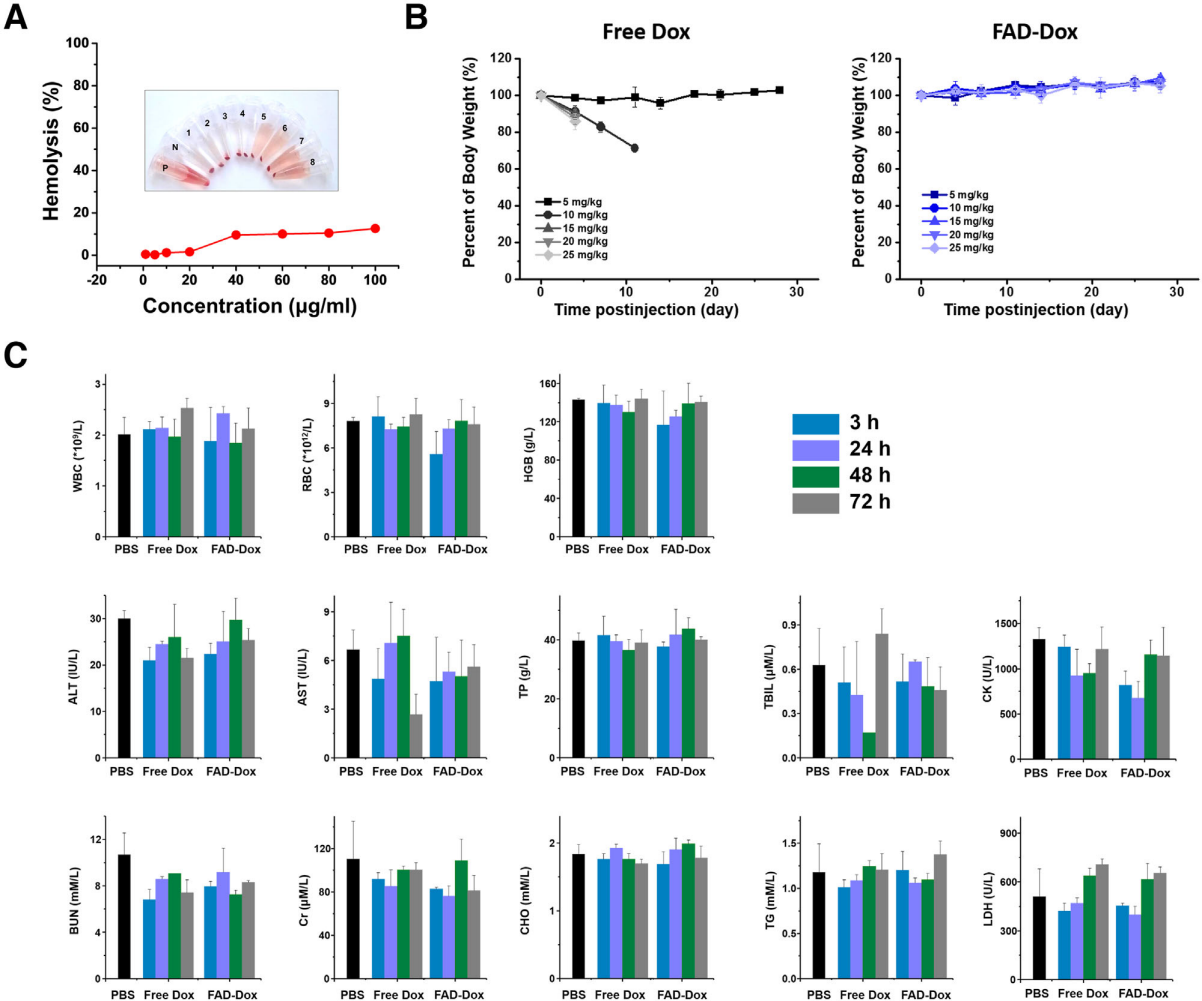
FAD‐Dox nanomicelles reduce the toxicity of doxorubicin in mice. (A) Hemolysis assay of empty FAD nanomicelles at different concentration. (B) Body weight change of mice after treatment with free Dox and FAD‐Dox. (C) Assessment of the in vivo toxicity of free Dox and FAD‐Dox nanomicelles in mice at different time‐points post‐injection by blood and biochemistry assays. WBC, white blood cells; RBC, red blood cells; HGB, hemoglobin; ALT, alanine aminotransferase; AST, aspartate aminotransferase; TP, total protein; TBIL, total bilirubin; BUN, urea nitrogen; Cr, creatinine; CHO, cholesterol; TG, triglyceride; CK, creatine kinase; LDH, lactate dehydrogenase

The toxicity of the FAD‐Dox nanomicelles was examined in the view of performing further biological studies in animal experiments. We first analyzed the blood compatibility of FAD‐Dox using mouse red blood cells in a hemolysis assay. Our results revealed that FAD‐Dox had negligible hemolytic effects at concentration below 40 µg/mL (Figure 4A), indicating the excellent blood compatibility of FAD‐Dox and its potential use for in vivo animal experiments. We then assessed the maximum tolerated dose (MTD) of FAD‐Dox. The MTD value is crucial for in vivo experimental design as it indicates the highest dose of a drug that does not cause unacceptable side effects. Accordingly, healthy mice were treated with repeated intravenous (i.v.) administration of the FAD‐Dox nanomicelles, using empty FAD nanomicelles, free Dox and PBS as controls. In the FAD‐Dox or FAD micelles treated group, we did not observe any morbidity, death, or weight loss in mice even at doses up to 25 mg/kg of Dox equivalent per mouse body weight (Figure 4B). This highlights the excellent safety profile of the FAD‐Dox nanodrug. In contrast, in the free Dox treated group at doses above 10 mg/kg, a significant body weight loss could be observed, and all the mice died after the first injection of free Dox at doses higher than 15 mg/kg. The corresponding MTD of free Dox was therefore fixed at 5.0 mg/kg, which was in accordance with previously reported studies.^[^ ^21^ ^]^ It is worth mentioning that the MTD of FAD‐Dox was higher than that of free Dox at more than fivefold change, which allows for a large window for dosage upscaling. As shown in Figure 4C, further blood biochemical analysis confirmed that there was no notable toxicity in any of the FAD‐Dox treated mice at 5.0 mg/kg, which is the MTD of free Dox. Altogether, these results demonstrate that the nanomicellar formulation of Dox using the FAD significantly reduced the acute and systemic toxicity of Dox, and highlights the power and advantages of nanotechnology‐based drug formulation.

### 3.4 FAD‐dox nanomicelles effectively inhibit and suppress tumor growth in PDX models

**FIGURE 5:**
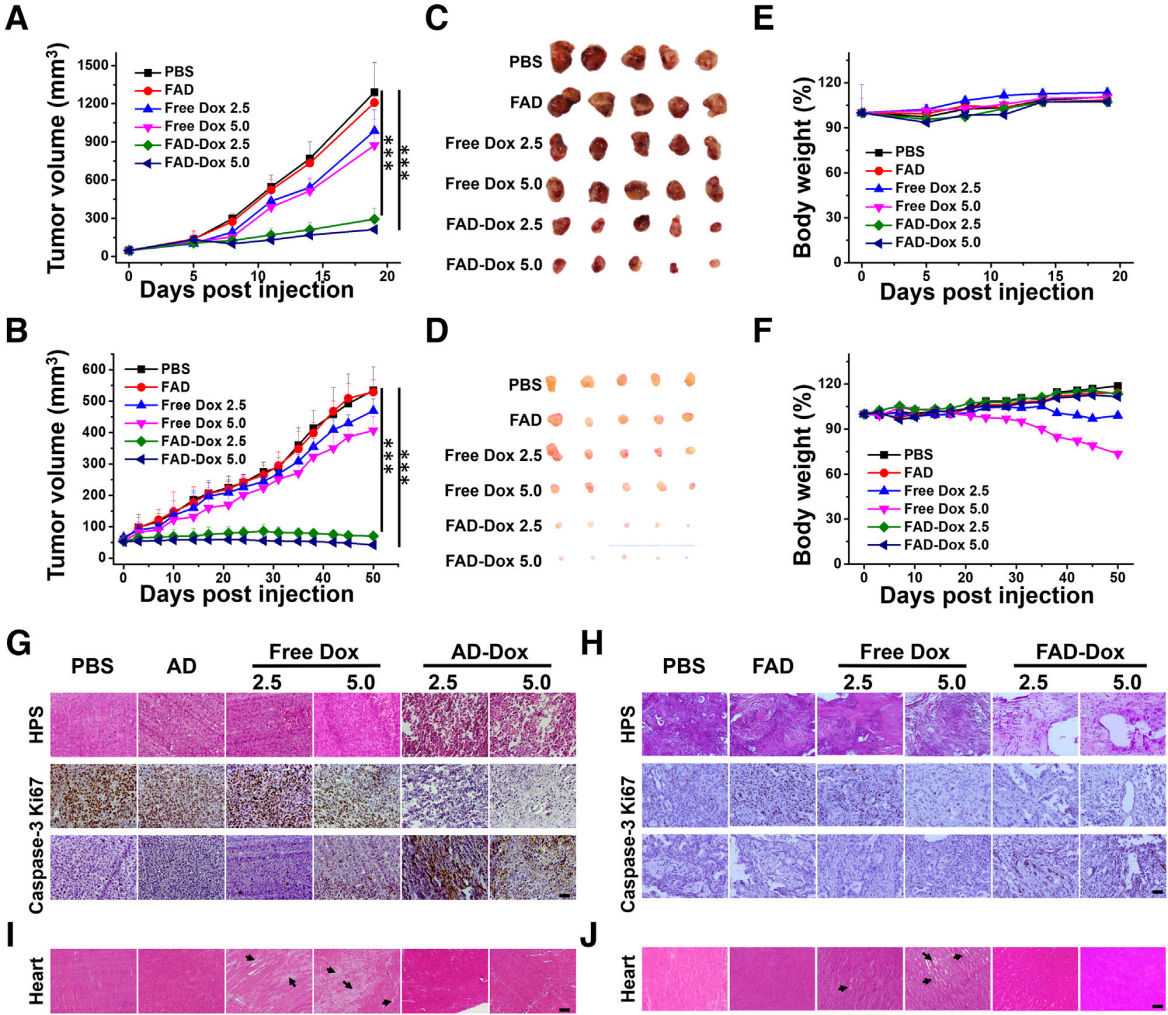
FAD‐Dox nanomicelles displayed effective anticancer activity without notable toxicity in patient‐tumor derived xenograft mice. Tumor growth in (A) PDAC087T and (B) PDAC074T xenograft mice, and representative images of excised tumors from (C) PDAC087T and (D) PDAC074T xenograft mice in the different treatment groups. Body weights of mice bearing (E) PDAC087T and (F) PDAC074T tumors were measured at different time‐points during the treatment regimes. HPS, Ki‐67, and Caspase‐3 immunohistochemical (IHC) staining of (G) PDAC087T and (H) PDAC074T tumor tissues after different treatments. Histological analysis of the hearts of mice bearing (I) PDAC087T and (J) PDAC074T tumors after different treatments. Scale bar: 20 µm. *p < 0.05; **p < 0.01; ***p < 0.001, two‐tailed Student's *t*‐tests

Encouraged by the in vitro antiproliferation activity and in vivo non‐toxic profile, we went on to evaluate the anticancer activity of FAD‐Dox nanomicelles using PDX mice generated from PDAC087T and PDAC074T cells, respectively. The mice were organized into different groups, based on the treatment received: (i) PBS buffer, (ii) FAD nanomicelles alone, (iii) free Dox at the dose of 2.5 mg/kg, (iv) free Dox at the dose of 5.0 mg/kg, (v) FAD‐Dox nanomicelles at the Dox equivalent dose of 2.5 mg/kg, and (vi) FAD‐Dox nanomicelles at the Dox equivalent does of 5.0 mg/kg. As we can see in Figure 5, the growth of PDAC087T and PDAC074T xenograft tumors in mice treated with FAD‐Dox nanomicelles was significantly inhibited (PDAC087T in Figure 5A,C and PDAC074T in Figure 5B,D and Figure S8). More importantly, in mice treated with FAD‐Dox at a high dose of 5.0 mg/kg tumor growth was almost suppressed. In contrast, the empty FAD nanomicelles did not affect the tumor growth and the results were similar to those observed for PBS‐treated mice. Treatment with free Dox exerted only a slight effect on tumor growth in both PDAC087T and PDAC074T xenograft mice. As the volumes of the PDAC087T xenograft tumors exceeded 1200 mm^3^, and the body weight of the PDAC074T xenograft mice was reduced by more than 20% after 50 days of treatment, we discontinued the animal experiments and sacrificed all mice according to ethical requirements. Immunohistochemical (IHC) analysis of PDAC087T and PDAC074T xenograft tumors (Figure 5G,H) demonstrated that the tumor cells showed necrotic changes. IHC analysis of the active (pro‐apoptotic) form of caspase‐3 revealed that cells with high caspase‐3 activity were much more in FAD‐Dox treated PDAC087T and PDAC074T tumor. Additionally, the reduced number of Ki‐67 positive tumor cells for, an indicator of proliferating cells, indicated that FAD‐Dox caused a considerable decrease in tumor cells’ proliferative activity. It is important to mention that the mice treated with PBS, free FAD, and FAD‐Dox showed no significant body weight loss, but rather a weight gain after a long period of treatment (Figure 5E,F). This finding is an additional indication of the superior safety profile of FAD‐Dox, as many anticancer drugs cause serious weight loss owing to their marked toxicity. In addition, no morphological changes or pathology in organs could be detected in FAD‐Dox‐treated mice (Figure 5I,J; Figure S9), whereas in the free Dox treated mice, myocardial fiber breakage and hyperemia of the heart were observed, which is consistent with the previously reported cardiotoxicity associated with Dox.^[^ ^29^ ^]^ In summary, FAD‐Dox allowed delivery of Dox with improved anticancer efficacy yet no observable toxicity, and with a superior therapeutic index when compared to the free drug Dox.

### 3.5 FAD‐dox nanomicelles are endowed with favorable pharmacokinetics and EPR‐mediated tumor targeting

**FIGURE 6:**
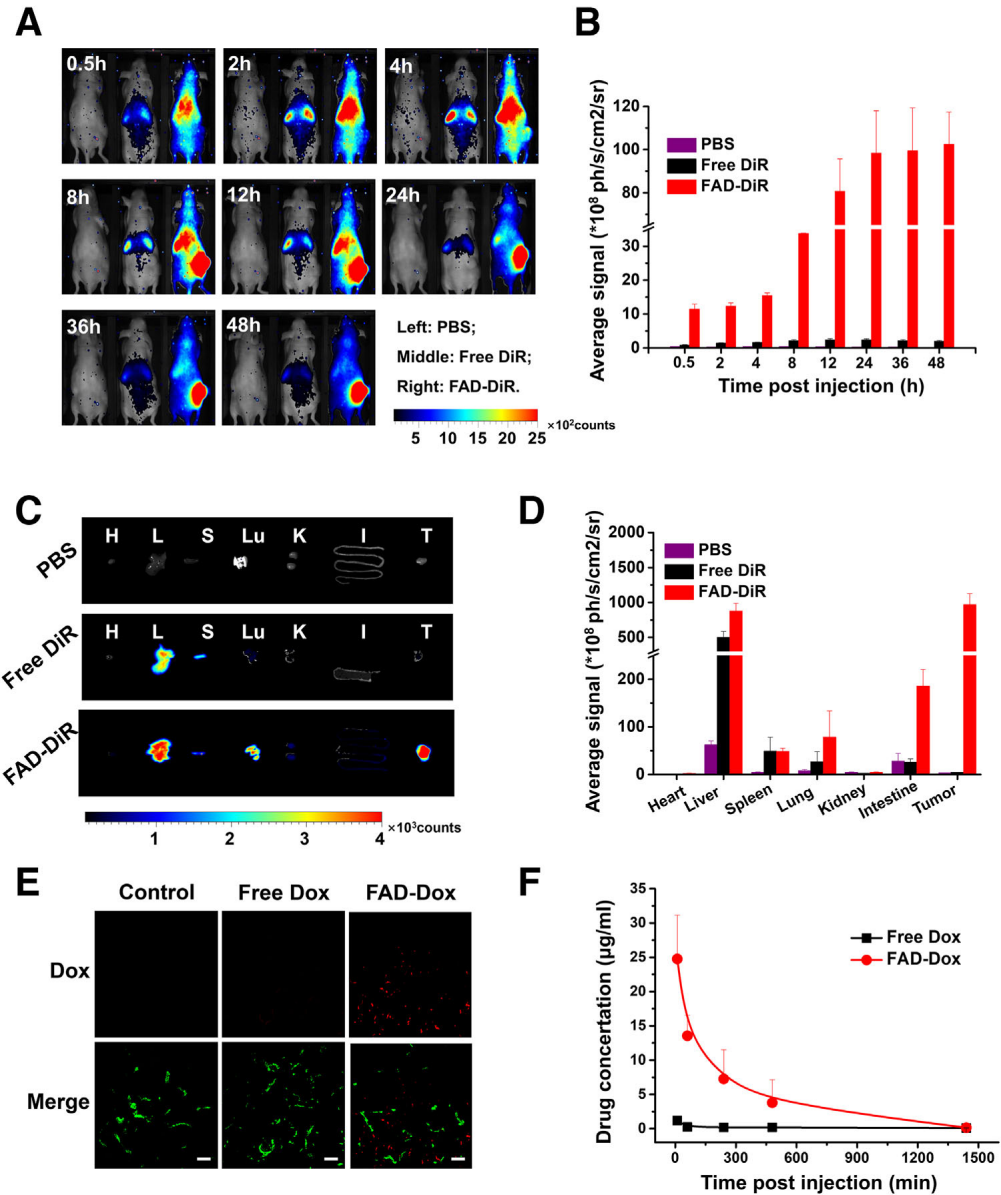
Biodistribution of the FAD‐Dox nanomicelles in mice. (A) In vivo images of mice with PDAC087T xenograft tumors at 0.5, 2, 4, 8, 12, 24, 36, and 48 h after treatment with PBS (left), free DiR (middle) or FAD‐DiR (right). (B) Average fluorescence signals of PDAC087T xenograft tumors at 0.5, 2, 4, 8, 12, 24, 36, and 48 h after treatment with PBS, free DiR, or FAD‐DiR. (C,D) Images (C) and average fluorescence signals (D) of heart, liver, spleen, lung, kidney, intestine, and tumor. Tissues were removed at 48 h post‐injection for ex vivo imaging, and analysis. (E) Penetration of Dox into the tumors of mice with PDAC087T xenografts. Mice were treated with free Dox and FAD‐Dox nanomicelles. Tumors were imaged by confocal microscopy. Scale bar: 10 µm, Red: Dox, Green: CD31 marker to indicate the blood vessels. (F) Plasma pharmacokinetics study of free Dox and FAD‐Dox nanomicelles in mice. The FAD‐Dox (red) prolonged the drug circulation in blood, compared to free Dox (black)

Motivated by the excellent in vivo anticancer activity of the FAD‐Dox nanomicelles, we wanted to further examine the biodistribution and pharmacokinetic behavior, which is important for the drug delivery efficiency and in vivo toxicity, and is also essential for the interpretation, understanding, and assessment of drug efficiency and safety. We examined the biodistribution of FAD nanomicelles in PDAC087T xenografts by loading DiR (FAD‐DiR), which is a near‐infrared fluorescent dye. Figure 6A shows the tumor accumulation and distribution of FAD‐DiR in real‐time at 0.5, 2, 4, 8, 12, 24, 36, and 48 h post‐injection. After 0.5 h, strong fluorescence could be catched in the entire animal body. Then the fluorescence could accumulate in the tumor site in the mice with FAD‐DiR treatment after 8 h. Indeed, the average fluorescence signal at the tumor site continued to increase in mice treated with FAD‐DiR and was always much higher than that measured in mice treated with free DiR (Figure 6B). These results imply that FAD‐DiR nanomicelles were able to alter the biodistribution of Dox by enhancing the drug accumulation at tumor site while sparing healthy organs the negative effects of drug toxicity (Figure 6C,D). In addition, thanks to their small size of ∼10 nm, the FAD‐Dox nanomicelles not only accumulated at the tumor site more efficiently but also penetrated deeper and spread further around the blood vessels than free Dox, as demonstrated in Figure 6E. We also generated 3D cultured multicellular spheroids from PDAC087T and PDAC074T cells and treated them with FAD‐Dox and Dox. Biphoton microscopy imaging also confirmed that FAD‐Dox penetrated more deep in the cancer cell spheroids (Figure S10). Together, these results confirm that FAD‐Dox nanomicelles have longer blood circulation, higher tumor accumulation, and deeper tumor penetration efficiency than free Dox. The FAD‐Dox nanomicelles hence generate a higher local drug concentration at tumor and are able to circumvent heterogeneous chemo‐resistance and have low systemic toxicity.

To examine the pharmacokinetic behavior, we administered healthy mice with FAD‐Dox and free Dox via i.v. and analyzed the Dox concentration in plasma samples. Free Dox was rapidly cleared from the blood, while FAD‐Dox led to a considerably increased Dox concentration and blood circulation time compared to the free anticancer drug (Figure 6F). The terminal blood elimination half‐life of Dox in blood plasma was increased to 135 min for FAD‐Dox compared to 21 min for free Dox, about a 6.5‐fold enhancement. The AUC (area under the concentration/time curve) level increased from 51 to 3918, an increase of almost 77‐fold. The significantly prolonged plasma half‐life and increased AUC demonstrate that FAD‐Dox improves drug retention and facilitates time‐dependent drug accumulation in tumors, in particular via the EPR effect for passive tumor targeting.

## 4 DISCUSSION

PDAC is a disease with considerable molecular heterogeneity and tumor microenvironment characterized by disorganized blood vessels and dense stroma, generating both biological and physical barriers that make PDAC extremely hard‐to‐treat. Nanomedicine, nanosized drug delivery systems with tailored features, represents a valuable strategy in pancreatic cancer therapy by virtue of their ability to combat various biological and physical barriers, namely, accumulating effectively at tumor site through EPR effect, penetrating deeply in tumor tissue by tailored small size, interacting with and entering into cancer cells via endocytosis for boosting local drug concentration in tumor, hence enhancing drug efficacy while overcoming drug resistance and heterogeneous responses and, at the same time, reducing drug toxicity.

Various nanodrug candidates have been developed and explored for treating PDAC, with MM‐398 and nab‐paclitaxel approved by FDA for use in combination therapies. However, both nab‐paclitaxel and MM‐398 monotherapies did not show any statistical improvement compared to current standard therapy. In this work, we elaborated an innovative dendrimer nanodrug system, which carry Dox with an encapsulation efficiency and a high drug loading. By virtue of small nanosize, stable formulation, long circulation time, and acid‐promoted drug release, this dendrimer nanosystem effectively accumulated at tumor with deep penetration and rapid drug uptake/release profile in the cancer cells, leading to potent anticancer activity and complete suppression of tumor growth in patient‐derived pancreatic tumor xenograft models. In addition, this dendrimer nanodrug formulation showed excellent tolerability and was devoid of any acute toxicity. This simple and convenient dendrimer nanodrug system holds great promise as a therapeutic candidate for further PDAC translational investigations.

It is also to mention that drug development for PDAC has been largely hampered because of the molecular heterogeneity of PDAC and the resulting discrepancies between outcomes of clinical trials and the drug effect obtained in preclinical study. The inconsistency can be attributed to the limitation of clinically relevant cancer models for preclinical drug evaluation. Primary human cancer cell lines obtained directly from individual patients can closely reflect the actual tumor situation, hence generating high‐fidelity data, offering good models for assessing the effectiveness of therapy prior to clinical validation.^[^ ^30^, ^31^ ^]^ In this study, we selected 35 primary human pancreatic cancer cells derived from patients, from the PaCaOmics cohort (clinical trial registration number NCT01692873),^[^ ^26^, ^27^ ^]^ to evaluate anticancer activity of our dendrimer nanodrug system FAD‐Dox. These primary cells displayed significant heterogeneity in their responses to common clinical anticancer drugs.^[^ ^28^ ^]^ Remarkably, all the primary cancer cells had a consistent response to our FAD‐Dox nanomicelles, which resulted in effective and homogenous inhibition of cell proliferation, with the superior performance of FAD‐Dox compared to the clinical drugs Dox and Caelyx, a Dox nanoformulation. Most importantly, our data demonstrated the consistently favorable response of all the 35 primary cancer cells to the FAD‐Dox nanomicelles, with IC_50_ values far below 10 µg/mL, highlighting that FAD‐Dox can markedly overcome the heterogeneity of drug response and drug resistance of primary cultured tumor cells derived from PDAC patients. In addition, FAD‐Dox completely suppressed tumor growth in patient‐derived pancreatic tumor xenografts without any notable adverse effects. Given all these uniquely advantageous features, this simple and convenient dendrimer nanodrug system holds great promise as a therapeutic candidate to treat the deadly PDAC, for which efficacious treatment is desperately sought‐after.

## CONFLICT OF INTEREST

The authors declare no conflict of interest.

## ETHICS STATEMENT

All the primary cells were derived from the PDAC patients were recruited for this study under the Paoli Calmettes Institute clinical trial number 2011‐A01439‐32. All the patient's informed consent had been obtained. And animal experiments were performed in agreement with the Animal Ethics Committee of the Bouche du Rhône prefecture in France.

## AUTHOR CONTRIBUTIONS

Ling Peng coordinated the project. Juan Liu, Ling Peng, and Xing‐Jie Liang conceived and designed the research. Chao Chen performed the synthesis of dendrimer. Juan Liu and Tuo Wei prepared the nanomicelles, and performed the evaluation and characterization. Odile Gayet, Laurence Borge, Nelson Dusetti, and Juan Iovanna supplied the primary cancer cells. Céline Loncle, Nelson Dusetti, and Juan Iovanna helped to establish the PDX model. Xiaowei Ma and Zhongwei Gu discussed and revised the manuscript. Domenico Marson, Erik Laurini, and Sabrina Pricl performed the ITC experiment and analyzed the results. Juan Liu, Sabrina Pricl, Ling Peng, and Xing‐Jie Liang discussed and wrote the manuscript.

## Supporting information

SUPPORTING INFORMATION

## Data Availability

All data associated with this study are present in the paper or in the Supporting Information.
