# Supplementary material for: Dendrimeric nanosystem consistently circumvents heterogeneous drug response and resistance in pancreatic cancer
Source: Exploration (Beijing). 2021 Aug 27;1(1):21–34. doi: 10.1002/EXP.20210003 (PMC10291567; doi:10.1002/EXP.20210003)
Supplement: Supplementary file 1 — SUPPORTING INFORMATION [file EXP2-1-21-s001.docx]

**Dendrimeric Nanosystem Consistently Circumvents Heterogeneous Drug Response and Resistance in Pancreatic Cancer**

Juan Liu, Chao Chen, Tuo Wei, Odile Gayet, Céline Loncle, Laurence Borge, Nelson Dusetti, Xiaowei Ma, Domenico Marson, Erik Laurini, Sabrina Pricl, Zhongwei Gu, Juan Iovanna, Ling Peng*, Xing-Jie Liang*

[*Companies (and their location) providing equipment, reagents and/or supplies* 3](#_Toc56796659)

[*Synthesis of amphiphilic dendrimers* 3](#_Toc56796660)

[*Critical micelle concentration.* 6](#_Toc56796661)

[*Isothermal titration calorimetry (ITC) experiments and computational methodology* 7](#_Toc56796662)

[*PDAC samples and cell culture* 9](#_Toc56796663)

[*Hemolysis assay* 10](#_Toc56796664)

[*Tolerability of FAD-Dox in healthy mice* 11](#_Toc56796665)

[*Blood chemistry analysis* 11](#_Toc56796666)

[*Tissue histology* 11](#_Toc56796667)

[*Immunohistochemistry assay* 12](#_Toc56796668)

[*Drug penetration study in 3D-cultured tumor spheroids* 12](#_Toc56796669)

[Table S1. 14](#_Toc56796670)

[Table S2. 15](#_Toc56796671)

[Table S3. 16](#_Toc56796672)

[Figure S1. 17](#_Toc56796673)

[Figure S2. 18](#_Toc56796674)

[Figure S3. 19](#_Toc56796675)

[Figure S4. 21](#_Toc56796676)

[Figure S5. 22](#_Toc56796677)

[Figure S6. 23](#_Toc56796678)

[Figure S7. 24](#_Toc56796679)

[Figure S8. 25](#_Toc56796680)

[Figure S9. 26](#_Toc56796681)

[Figure S10. 27](#_Toc56796682)

[References 28](#_Toc56796683)

# *Companies (and their location) providing equipment, reagents and/or supplies*

Abcam, Cambridge, MA; Analyzer Medical System, Roma, Italy; BD Biosciences, Franklin Lakes, NJ; Carl Zeiss Microscopy, Oberkochen, Germany; Charles River Laboratories, Wilmington, MA; FEI, Hillsboro, OR, USA; Gibco and Invitrogen, Life Technologies Corp., Grand Island, NY, USA; Hitachi, Tokyo, Japan; Leica, Washington, DC; Lonza Inc., Walkersville, MD; Malvern, Worcestershire, U.K.; Miltenyi Biotec, Bergisch Gladbach, Germany; Merck Millipore, Billerica, MA, USA; Tecan, Durham, USA; Sigma-Aldrich, München, Germany and also St. Louis, MO; Perkin-Elmer Instruments Co., Ltd., USA; Vector Laboratories, Burlingame, CA, USA.

# *Synthesis of amphiphilic dendrimers*

All the reagents and solvents were purchased from Sigma Aldrich or Alfa Aesar. Methyl acrylate and ethylenediamine were distilled before use. Anhydrous THF was distilled in the presence of sodium-benzophenone with a deep blue color being persisting. All other reagents and solvents of analytical grade were used as received from commercial sources without further purification. ^1^H NMR spectra were recorded at 250, 400 or 500 Hz, ^13^C NMR spectra recorded at 62.5, 100 or 125 MHz. ^19^F NMR spectra were recorded at 235, 376 or 470 MHz on Bruker Avance II 250, 400 and 500 spectrometers (Bruker, Wissembourg, France), respectively. Coupling constants (*J*) are reported in Hertz, and chemical shifts are reported in parts per million (ppm).

High resolution mass spectrometry (HRMS) experiments were performed using a QStar Elite mass spectrometer (Applied Biosystems SCIEX, Concord, ON, Canada) or SYNAPT G2 HDMS mass spectrometer (Waters, Guyancourt, France) equipped with an electrospray ionization source operated in the positive mode.

Infrared spectra were recorded with an ALPHA FT-IR spectrometer (Bruker, Wissembourg, France). Compounds 2 and AD were synthesized according to the literature.[1] FAD were synthesized according to the Figure S1.

3：To the azide precursor 1(41.7 mg, 0.0912 mmol) were added CuSO_4_⋅5H_2_O (2.10 mg, 0.00829 mmol) sodium ascorbate (3.30 mg, 0.0166 mmol) and a solution of 2 (118.5 mg, 0.0829 mmol) in THF. The vessel was sealed and purged with argon for 5 min and H_2_O was then added into the mixture. The reaction mixture was stirred at 60 °C for 3 h until the reaction was completed, as indicated by TLC analysis. The THF was evaporated under reduced pressure and the resulting residue was suspended in EDTA solution. The water phase was extracted with CH_2_Cl_2_ (15 mL × 3). The combined organic layers were dried over MgSO_4_, filtered and concentrated to obtain a residue. The residue was purified by column chromatography on silica gel (EtOAc/MeOH = 3/1-2/1) to yield 3 as a colorless oil (132 mg, 82%).

^1^H NMR (400 MHz, CDCl3): δ 7.74 (br s, 2H, -NH-), 7.58 (s, 1H, -CH- triazole), 7.07-7.04 (m, 4H, -NH-), 4.32 (t, 2H, J = 9.8 Hz, -CH_2_-), 3.82 (s, 2H, -CH_2_-), 3.66 (s, 24H, -OCH_3_), 3.29-3.27 (m, 12H, -CH_2_-), 2.81-2.73 (m, 28H, -CH_2_-), 2.58-2.52 (m, 12H, -CH_2_-), 2.45-2.36 (m, 28H, -CH_2_-), 1.91-1.86 (m, 2H, -CH_2_-), 1.62-1.57 (m, 2H, -CH_2_-), 1.31-1.25 (m, 22H, -CH_2_-);13C NMR (100 MHz, CDCl_3_): δ 172.9, 172.1, 172.0, 143.5, 122.6, 53.3, 52.7, 52.3, 51.4, 51.4, 50.1, 49.7, 49.1, 47.2, 37.2, 37.0, 33.7, 33.4, 32.5, 30.5, 30.2, 29.4, 29.3, 29.2, 29.0, 28.9, 26.4, 19.8; ^19^F NMR (282 MHz, CDCl_3_/CD_3_OD = 3/1): -81.0, -114.6, -124.5, -126.1; HRMS: calcd. for C_83_H_143_F_9_N_16_O_22_^2+^ [M+2H]^2+^ 943.5204, found 943.5203.

4：To a solution of 3 (130 mg, 0.0689 mmol) in methanol was added ethylenediamine (1.50 mL, 22.4 mmol). The reaction mixture was stirred under argon for 24 h at 30 °C until the IR analysis showed the complete consumption of the ester starting material. The reaction solution was evaporated, and the obtained residue was purified by precipitation with CH_3_OH/Et_2_O three times, the obtained residue was purified by dialysis with a dialysis tube (MWCO=1000). Subsequent lyophilization yielded FDA 4 as white powder (133 mg, 91%).

^1^H NMR (300 MHz, CDCl3/CD3OD = 3/1): δ 7.74 (s, 1H, -CH- triazole), 4.33 (t, 2H, J = 7.4 Hz, -CH_2_-), 3.78 (s, 2H, -CH_2_-), 3.39-3.21 (m, 28H, -CH_2_-), 2.76-2.71 (m, 44H, -CH_2_-), 2.55-2.53 (m, 12H, -CH_2_-), 2.40-2.30 (m, 28H, -CH_2_-), 1.90-1.87 (m, 2H, -CH_2_-), 1.59-1.56 (m, 2H, -CH_2_-), 1.30-1.24 (m, 22H, -CH_2_-); 13C NMR (100 MHz, CD3OD): δ 175.2, 174.8, 174.6, 144.8, 125.2, 53.5, 53.4, 51.4, 51.2, 50.5, 42.9, 42.1, 38.6, 34.8, 31.6, 31.4, 30.8, 30.7, 30.6, 30.4, 30.2, 27.6, 21.3; 19F NMR (282 MHz, CDCl_3_/CD_3_OD = 3/1): -77.7, -111.1, -120.9, -122.6; HRMS: calcd. for C_91_H_176_N_32_O_14_F_9_^3+^ [M+3H]^3+^ 704.1295, found 704.1296.

# *Critical micelle concentration.*

The critical micelle concentrations of AD and FAD were first estimated by fluorescence measurement using pyrene as a fluorescent probe. The pyrene concentration in the solution was fixed at 6.0 × 10^−7^ M and the concentration of the dendrimer AD and FAD was varied from 3.0 × 10^−8^ to 5.0 × 10^−4^ M. The solutions were then sonicated for 30 min and kept for 2 h at room temperature to finalize the micelle formation. When the polymeric micelles were formed, pyrene was preferentially incorporated into the hydrophobic micelle core instead of the polar environment (aqueous solution). And the sharp rise in the intensity ratio (I_373_/I_384_) of pyrene in the excitation spectra indicates the onset of micellization for the amphiphilic dendrimer. Fluorescence excitation spectra (300 – 360 nm) of the solutions were recorded using an fluorescence spectrometer (Hitachi) at an emission wavelength of 335 nm, with the excitation and emission bandwidths set at 5 nm slit widths. The fluorescent intensity ratio of I_373_/I_384_ was analyzed as a function of logarithm AD or FAD concentration.

# *Isothermal titration calorimetry (ITC) experiments and computational methodology*

ITC experiments were performed with a MicroCal PEAQ-ITC calorimeter (Malvern, UK) at 25°C. The cell volume was 208 μL. All experiments were conducted in a forward manner, that is, by step-by-step injections of a constant volume of concentrated AD and FAD solutions into the calorimetric cell containing milliQ water. Specifically, for CMC determination, a constant 1 μL portion of each amphiphilic dendrimer solutions, at a concentration of 125 μM, was injected 37 times into the reaction cell at 210 s intervals. All solutions and buffer were degassed for 30 min at room temperature under stirring at 600 rpm prior to each experiment. After careful washing, the cell was pre-rinsed with a portion of the milli Q water and upon filling cell and syringe, stirring was turned and each system was allowed to thermally equilibrate for 30 minutes. All experiments were run in triplicates.

Figure 1G and H in the main text shows the demicellization thermograms of AD and FAD in milliQ water solutions. The injection of the concentrated AD or FAD solution into water mostly resulted in exothermic signals (inserts in Figure 1G and H): the large enthalpy change characterizing this first part of the thermogram, corresponding to the addition of the AD or FAD stock solution (which, for each system, was always higher than the corresponding CMC) to water, is due to demicellization, micelle dilution, and dilution of the surfactant monomers. When more micellar solution of AD or FAD is added to the sample cell, a clear decrease in the heat flow is observed indicating that the added micelles are not dissociated so the CMC is gradually approached at each injection. In the final part of the thermogram, the further addition of a concentrated AD or FAD micellar solution to the sample cell leads to heat effects due to micellar dilution, being AD or FAD concentration in the cell well above the corresponding CMC. Subtraction of the micelle dilution heat, normalization per mol of AD or FAD, and integration of the heat raw data yielded the observed heat Q as a function of Ad or FAD concentration.

The CMC values for AD and FAD are defined as the midpoint of the corresponding Q vs. C curve. For a precise determination of the midpoint of the demicellization process, first Q vs C data were fitted to a suitable model and then the first derivative of the Q vs C fitting curve was calculated (Figure S4A and B). The CMC corresponds to the maximum of the derivative curve, as highlighted by the arrow in Figure S4 A and B. From the ITC titration curve, the ΔH_mic_ is calculated from the enthalpy difference of the two levels of the titration curve as shown in Figure S4C for FAD as an example.

The values of the degree of counterion binding parameters β (Table S1) were obtained by measuring the CMC values of both AD and FAD micelles at 4 different counterion concentrations (2.5 μM, 5.0 μM, 7.5 μM, and 10 μM NaCl, respectively). By plotting ln CMC vs. ln [NaCl] for each micelle type, the corresponding β values could be obtained as the slope of the linear relation (known as the Corrin-Harkins relation), as shown in Figure S4D for FAD as an example.

The AD and FAD micellar aggregation numbers (N_agg_), tabulated in Table S1, were obtained from the simulation of the calorimetric titration curves on the basis of the mass action model by applying the same methodological approach described in detail by Olesen et al. [2].

# *PDAC samples and cell culture*

Two types of PDAC samples were obtained: endoscopic ultrasound-guided fine-needle aspiration (EUS-FNA) biopsy samples from patients with unresectable tumors, and tumor tissue samples from patients undergoing surgery. All the samples were anonymized, and postsurgical anatomopathology reports were provided for specimens from each patient. Each sample was mixed with 100 μL of Matrigel (BD Biosciences) and was injected in the upper right flank of a nude mouse (Swiss Nude Mouse Crl: NU(lco)-Foxn1nu; Charles River Laboratories). When the tumors reached 1 cm^3^, the mice were sacrificed, and the tumors were removed. The study on animals was approved by the Animal Facility and Experimental Platform (Scientific and Technological Park of Luminy, Marseille, France). Xenografts obtained from mice were split into several small pieces and used for cell culture, histologic analyses, reimplantation into new mice, and freezing. The fragments designated for cell culture were processed in a biosafety chamber: after fine mincing, they were treated with collagenase type V (Sigma-Aldrich) and trypsin/EDTA (Gibco) and were suspended in the modified medium. PDAC087T and PDAC070T cells were cultured in DMEM medium supplemented with 1% w/w penicillin/streptomycin (Gibco) and 10% fetal bovine serum (Lonza). Other cells were cultured in the DMEM/F12 medium containing 1.22 g/L nicotinamide, 5 g/L glucose, 5% Nu-Serum IV, 0.5% Insulin/Transferrin/Selenium Supplements, 1 μM dexamethasone, 10 ng/L cholera toxin, 50 nM 3,3’5-triiodo-L-Thyronine, 25.2 mg/L bovine pituitary extract, 20 μg/L epidermal growth factor. After centrifugation, cells were resuspended at 37^o^C in a 5% CO_2_ incubator. Amplified cells were stored in liquid nitrogen. Cells were weaned from antibiotics for 48 h before testing.

# *Hemolysis assay*

Red blood cells (RBCs) were isolated from 800 μL of freshly collected whole blood by centrifugation at 10000×g for 5 min, then washed five times with sterile isotonic PBS. RBCs were next suspended in 7.5 mL PBS and 0.5 mL of the resulting suspension were added to 0.5 mL of empty micelles suspended in PBS at the final concentration up to 100 μg/mL. 0.5 mL diluted RBC suspension incubated with 0.5 mL PBS was used as the negative control. The same amount of RBCs incubated with 1 mL water was used as the positive control. The samples were vortexed, left at room temperature for 3 h, then centrifuged at 10000×g for 5 min. 200 μL supernatant was transferred to a 96-well plate and the absorbance value of hemoglobin at 577 nm was measured with a reference wavelength of 655 nm. The percentage of hemolysis was calculated as follows:

Hemolysis % = [(sample absorbance – negative control) / (positive control – negative control)] ×100%

# *Tolerability of FAD-Dox in healthy mice*

Female NMRI healthy mice (6–8 weeks of age) were randomly assigned to the following treatment groups (n = 6 per group): free Dox or FAD-Dox nanomicelles at Dox concentration of 5, 10, 15, 20, and 25 mg/kg. All treatments were administrated via tail vein injection twice a week, and all drugs were diluted with PBS and injected in a single dose. Body weight and clinical observations were monitored daily until 28 days after injection.

# *Blood chemistry analysis*

Blood and serum was sampled from mice administered with free Dox or FAD-Dox nanomicelles at regular intervals of 3, 24, 48, and 72 h for blood chemistry analysis using a SABA-18 automatic biochemical analyzer (Analyzer Medical System).

# *Tissue histology*

All tissues, including tumor, heart, liver, spleen, lung, kidney, and intestine, were excised, imaged and fixed by immersion in a 4% paraformaldehyde solution for 24 h. The fixed tissue grafts were bisected and paraffin embedded. Serial 5 µm sections were obtained from each of the paraffin embedded wounds using a Leica microtome. Sections were collected and stained with hematoxylin and eosin stain (Sigma) according to the manufacturer’s protocols. Images of the stained samples were captured by a light microscope (Vectra® 3, PerkinElmer).

# *Immunohistochemistry assay*

Briefly, paraffin sections were rehydrated, incubated in an antigen retrieval solution and blocking serum, and stained using primary antibodies to Ki67, and caspase3 (Abcam), respectively. Subsequently, the sections were incubated with the universal secondary antibody (VECTOR) and VECTASTAIN Elite ABC reagent, washed in PBS, and reacted with ImmPACT DAB enzyme substrate. Finally, the sections were counterstained with hematoxylin and mounted using antifade mounting medium. Images of the stained samples were also captured by a light microscope (Vectra® 3, PerkinElmer).

# *Drug penetration study in 3D-cultured tumor spheroids*

Multicellular tumor spheroids were constructed with PDAC087T and PDAC074T cells using the liquid overlay method. The cells were seeded in round-bottomed 96-well plates with an ultralow attachment surface (Costar), with culture medium partially replaced (100 μL) by fresh medium every other day. To evaluate the drug penetration ability, the spheroids were incubated with free Dox or FAD-Dox nanomicelles at a Dox concentration of 10 μg/mL for 4 h. Then the tumor spheroids were washed twice with PBS and fixed by 4% (wt/vol) formaldehyde solution for 20 min, followed by further extensive (3x) washing. The fixed spheroids were transferred to confocal dishes and analyzed using a two-photon microscope (Carl Zeiss LSM7MP). Z-stack images were obtained by scanning the tumor spheroids step by step with 10 μm thickness. This experiment was performed at the PiCSL-France-BioImaging core facility (Institut de Biologie du Développement de Marseille, Aix-Marseille Université), a member of the France-BioImaging national research infrastructure.

# Table S1.

Drug loading content and drug encapsulation efficiency of Dox-loaded micelles.

| Dendrimer/ Dox ratio (w/w) | AD-Dox Encapsulation efficiency (%) | AD-Dox Drug loading (%) | FAD-Dox Encapsulation efficiency (%) | FAD-Dox Drug loading (%) |
| --- | --- | --- | --- | --- |
| 15:4 | 99.4 ± 1.07 | 20.9 ± 0.17 | 92.2 ± 3.43 | 19.72± 0.64 |
| 15:6 | 96.6 ± 1.02 | 27.9 ± 0.21 | 88.9 ± 1.32 | 26.2 ± 0.36 |
| 15:8 | 90.3 ± 0.32 | 32.5 ± 0.08 | 82.2 ± 1.25 | 30.5 ± 0.33 |
| 15:10 | 92.3 ± 1.24 | 38.1 ± 0.32 | 79.8 ± 2.17 | 34.7 ± 0.62 |
| 15:12 | 91.9 ± 2.63 | 42.4 ± 0.70 | 71.9 ± 1.16 | 36.5 ± 0.43 |
| 15:14 | 82.8 ± 1.48 | 43.6 ± 0.44 | 66.6 ± 0.62 | 38.3 ± 0.27 |
| 15:16 | 74.8 ± 1.24 | 44.4 ± 0.41 | 64.8 ± 0.20 | 40.9 ± 0.18 |
| 15:20 | 64.8 ± 1.08 | 46.3 ± 0.41 | 62.6 ± 3.04 | 45.4 ± 1.20 |
| 15:25 | 60.4 ± 0.16 | 50.2 ± 0.07 | 55.3 ± 2.05 | 47.9 ± 0.91 |
| 15:30 | 57.8 ± 0.88 | 53.6 ± 0.38 | 44.6 ± 0.21 | 47.2 ± 0.15 |

# Table S2.

AD and FAD micellization thermodynamic data (standard deviation in parenthesis) as obtained from ITC experiments (see SI text for details). Experimental results are expressed as mean of three experiments ± standard deviation. β = degree of counterion binding parameter. N_agg_ = micelle aggregation number.

|  | CMC (μM) | ΔH_mic_ (kcal/mol) | ΔG_mic_ (kcal/mol) | TΔS_mic_ (kcal/mol) |  | N_agg_ |
| --- | --- | --- | --- | --- | --- | --- |
| AD | 11.2 ± 0.9 | 19.23 ± 0.38 | -10.33 | 29.56 | 0.67 | 10.4 ± 0.1 |
| FAD | 6.01 ± 0.15 | 19.98 ± 0.32 | -10.92 | 30.90 | 0.68 | 9.7 ± 0.2 |

# Table S3.

Thermodynamic data for the interactions of AD and FAD nanomicelles with Dox as extracted from ITC analysis. Experimental results are expressed as mean of three experiments ± standard deviation. K_d_ = dissociation constant.

|  | pH | K_d_ (μM) | ΔH (kcal/mol) | ΔG (kcal/mol) | TΔS (kcal/mol) |
| --- | --- | --- | --- | --- | --- |
| AD | 5.0 | 6700 ± 230 | 3.31 ± 0.25 | -2.97 | 6.28 |
| FAD | 5.0 | 5400 ± 360 | 3.27 (0.22) | -3.09 | 6.36 |
| AD | 7.4 | 213 ± 29 | 7.95 (0.23) | -5.01 | 12.96 |
| FAD | 7.4 | 70 ± 15 | 8.21 (0.19) | -5.68 | 13.89 |

# Figure S1.

Synthesis pathway leading to the amphiphilic dendrimers AD (left) and FAD (right).


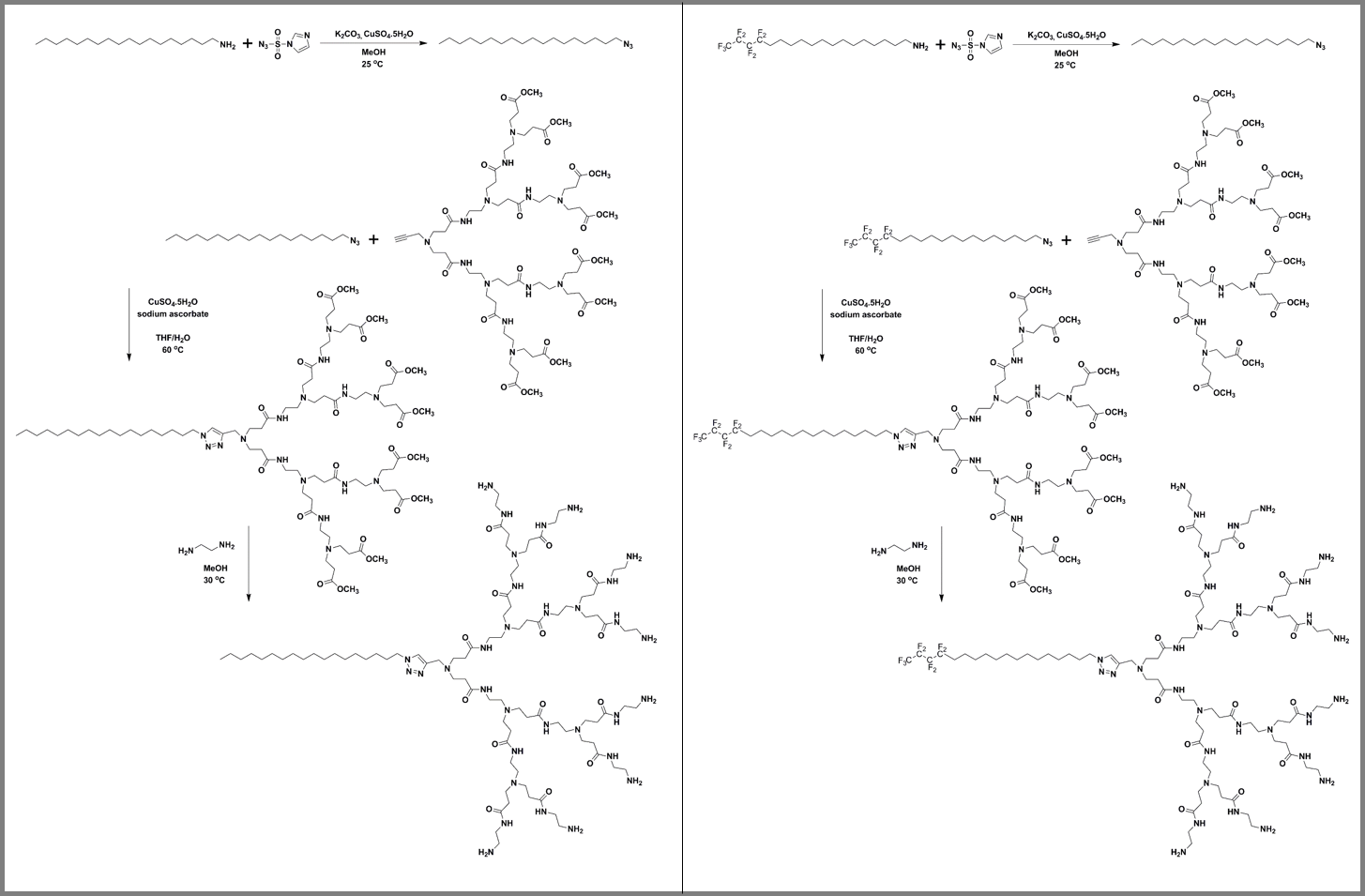


# Figure S2.

The critical micelle concentration of AD and FAD determined using the fluorescent dye pyrene at pH 7.4.


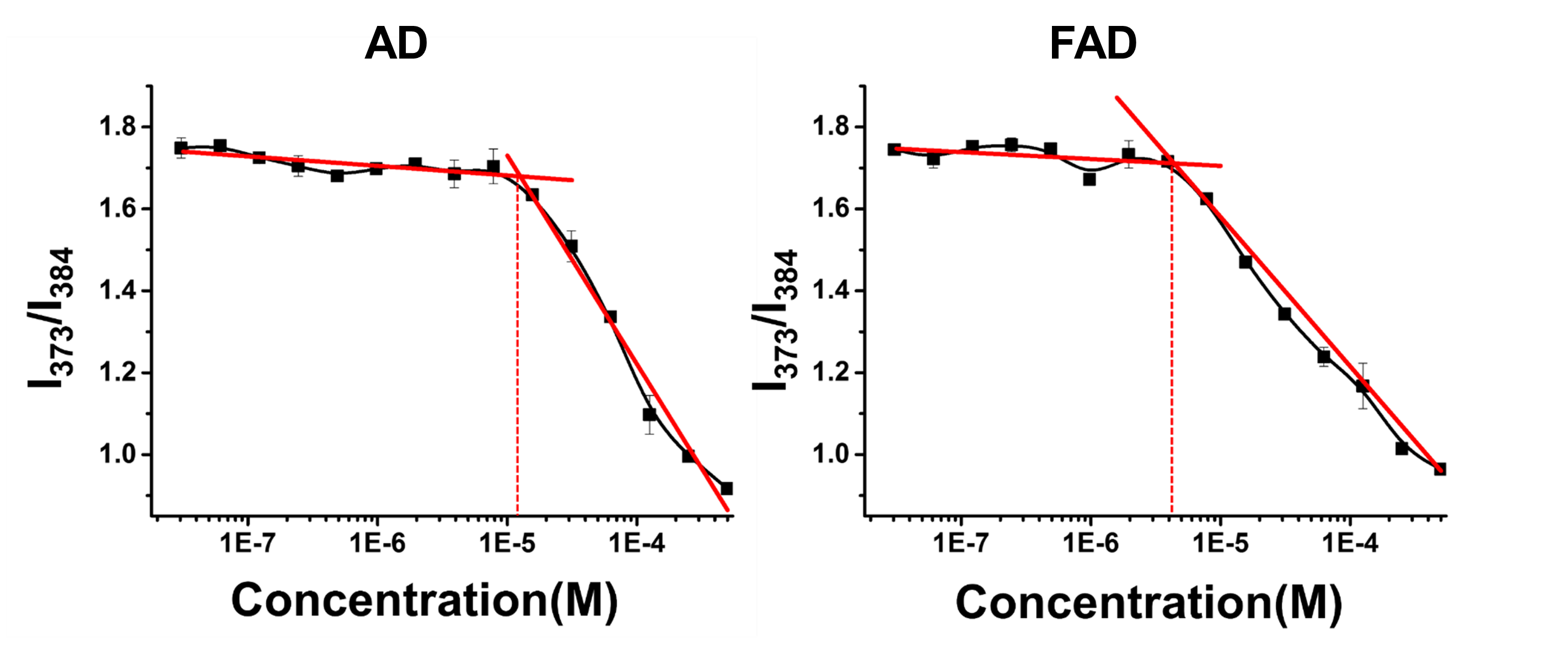


# Figure S3.

(A, B) Representative integrated isothermal calorimetry (ITC) profiles for the demicellization process of AD (A) and FAD (B) in milliQ water. The solid lines are data fitting with a sigmoidal function. The inserts in each panel show the corresponding ITC raw data. (C, D) Molecular dynamics simulation of AD (C) and FAD (D) spontaneous micelle formation shown at the initial, intermediate, and final simulations steps. AD and FAD molecules are shown as cyan and purple spheres, respectively.

**
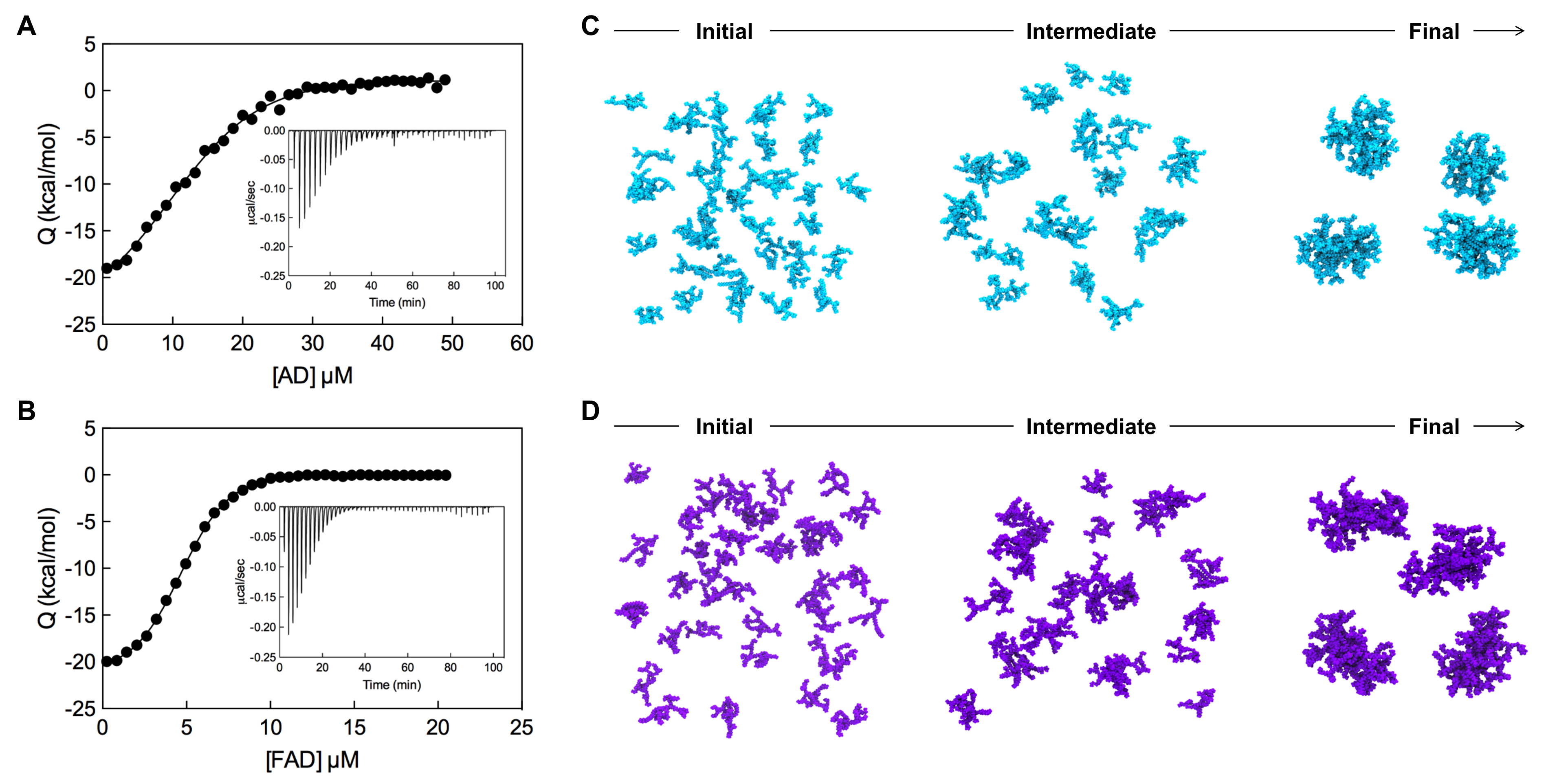
**

# Figure S4.

First derivative of the Q vs. C curves shown in Fig. 1G and H of main text, required to estimate the CMC values for AD and FAD by ITC. For each system the CMC locates at the maximum of the corresponding derivative curve, as indicated by the red arrows for AD (A) and FAD (B), respectively. (C) The ITC titration curve of FAD; the corresponding value of ΔH_mic_ is calculated from the enthalpy difference of the two plateau levels of the titration curve. (D) Plot of ln CMC vs. ln [NaCl] for FAD micelles, from which the corresponding value of the degree of counterion binding is obtained as the slope of the linear relationship.


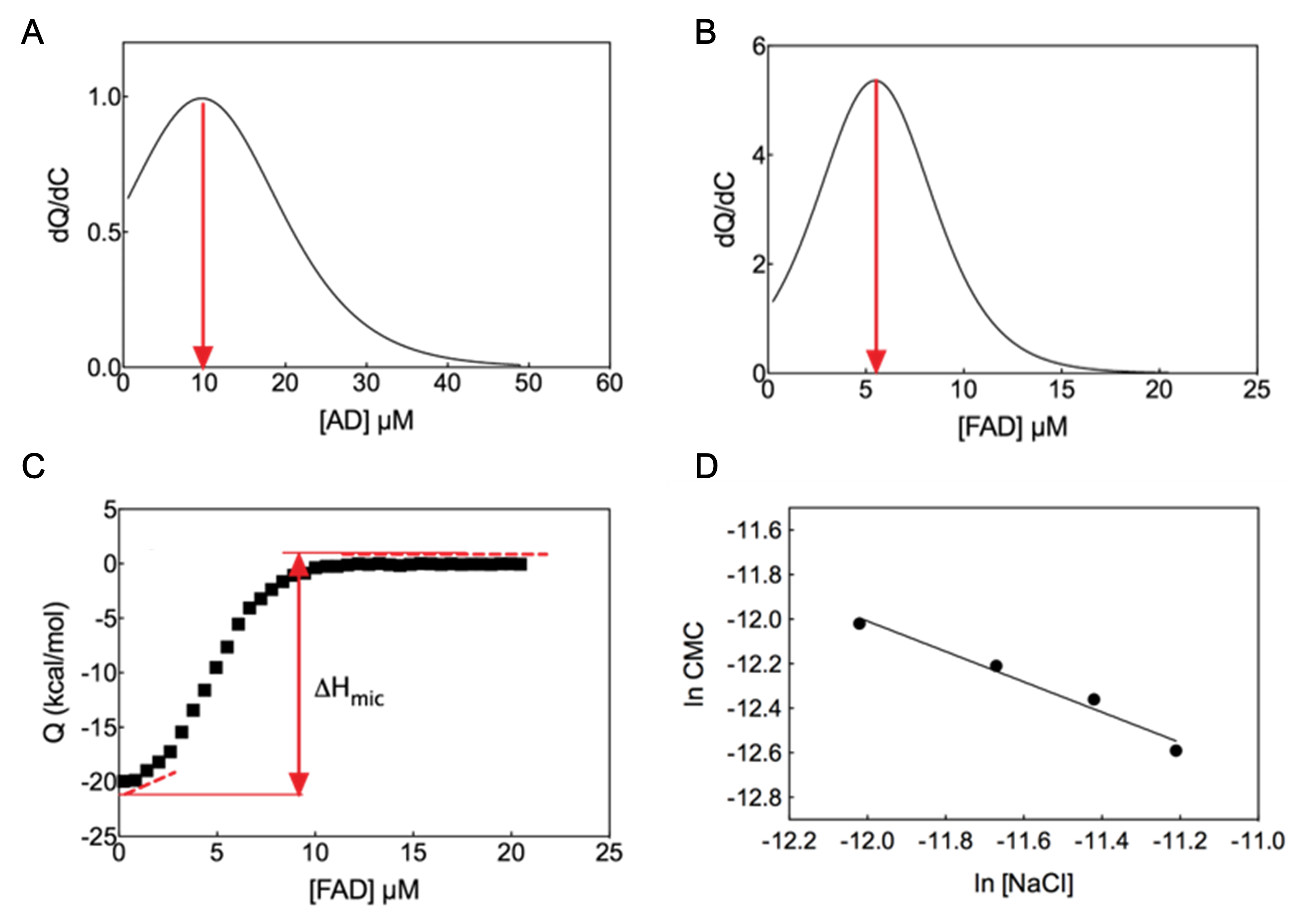


# Figure S5.

(A) TEM images of empty AD and FAD nanomicelles. (B) Size distribution of empty AD and FAD nanomicelles. (C) Size distribution of AD-Dox and FAD-Dox nanomicelles at Day 0 and Day 180.


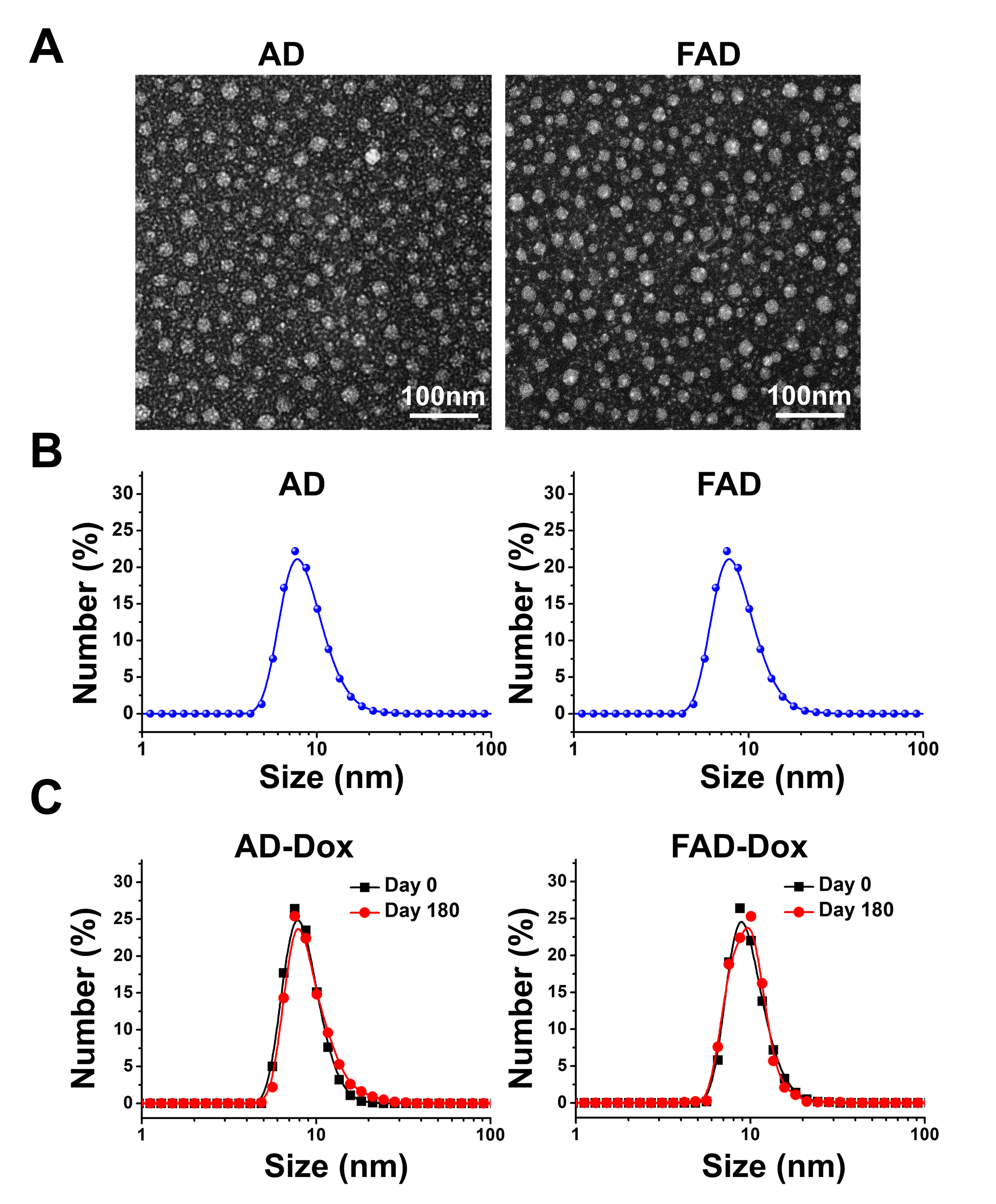


# Figure S6.

(A) Survival time of 35 pancreatic ductal adenocarcinoma patients. The red dashed line indicates the survival time division between short- and long-term survivor is 8 months. (B) Bright field images of the 35 primary cell lines.


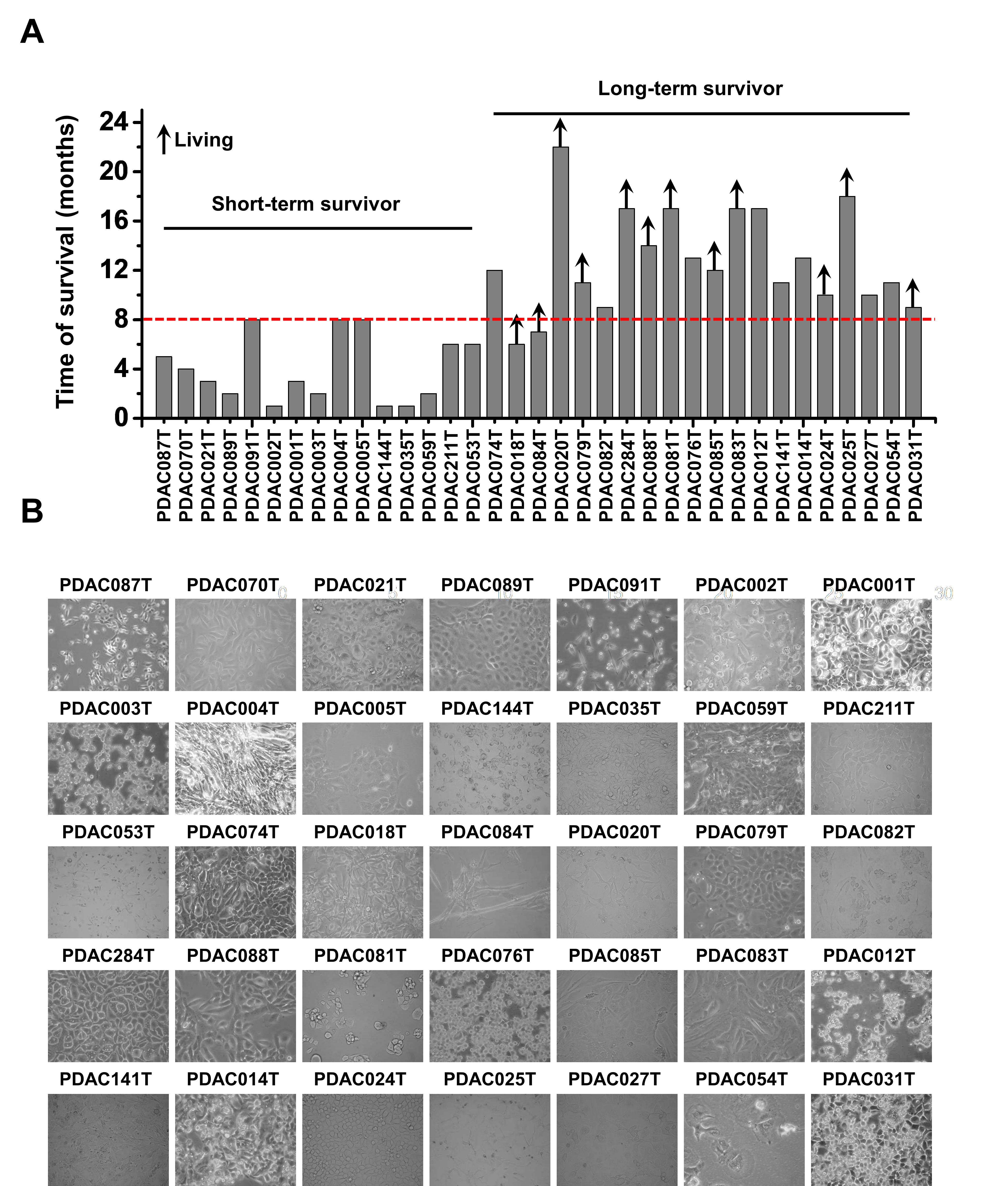


# Figure S7.

Mean fluorescence intensity of Dox in eight pancreatic cancer cells treated with free Dox and FAD-Dox nanomicelles for 1 h (A) and 4 h (B).


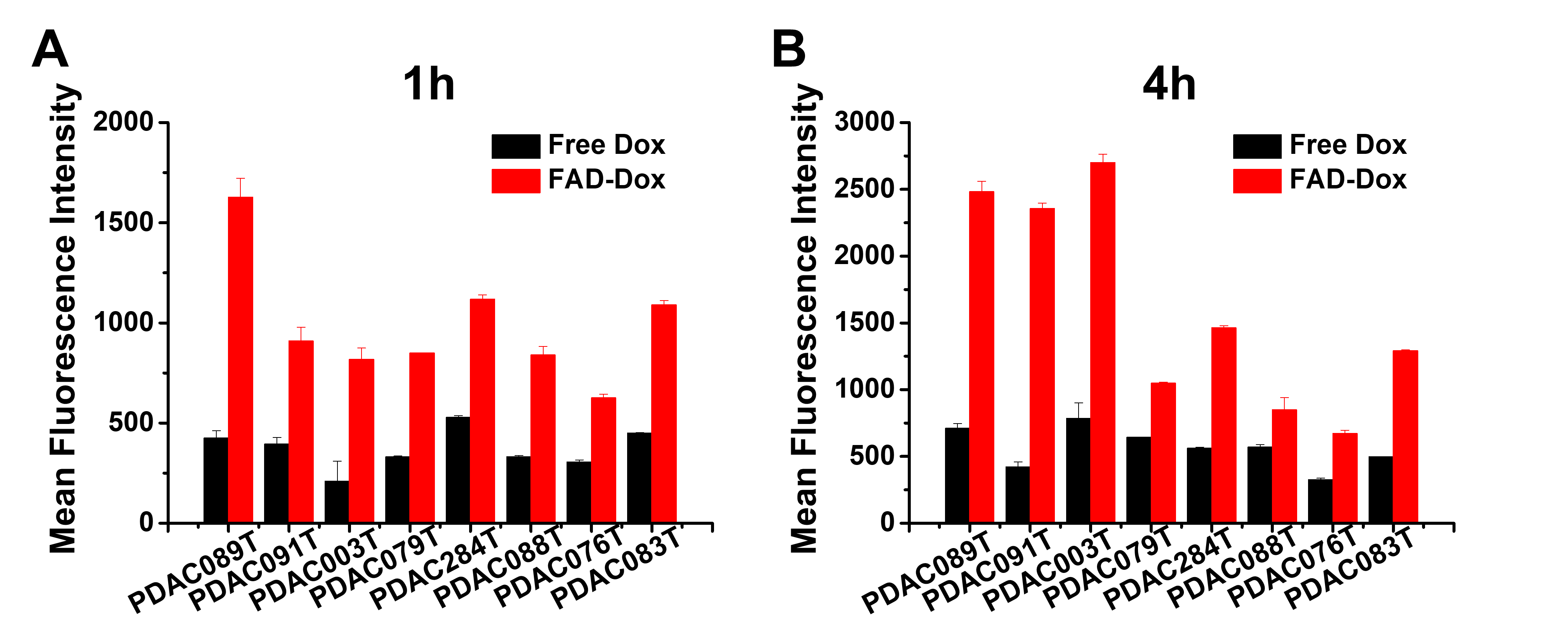


# Figure S8.

Weight of tumors excised from mice in different treatment groups.


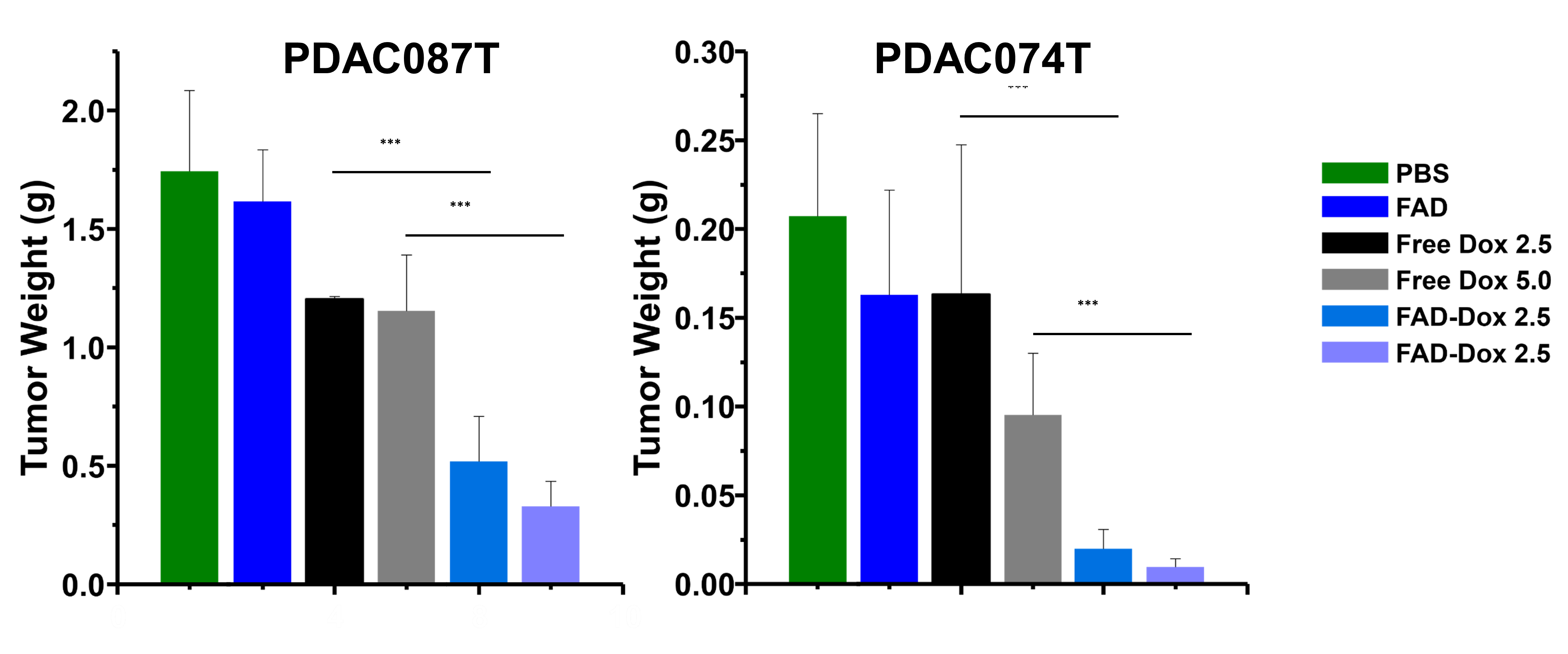


# Figure S9.

Histological analysis of liver, spleen, lung, kidney and intestine of PDAC087T and PDAC074T xenografts after different treatments.


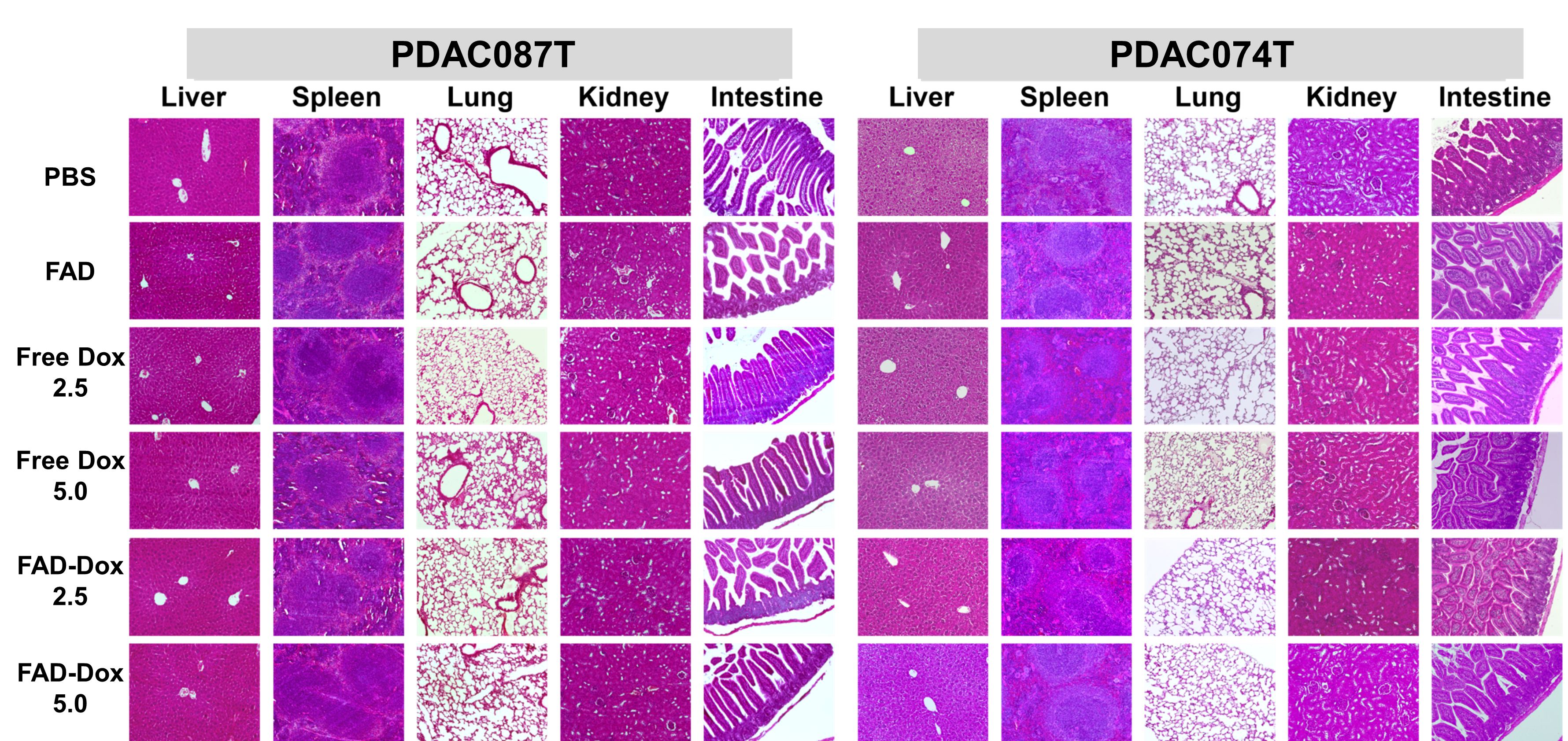


# Figure S10.

Penetration of Dox into 3D-cultured PDAC087T (A) or PDAC074T (B) tumor spheroids treated with free Dox and FAD-Dox nanomicelles. Images were obtained by two-photon confocal microscopy.


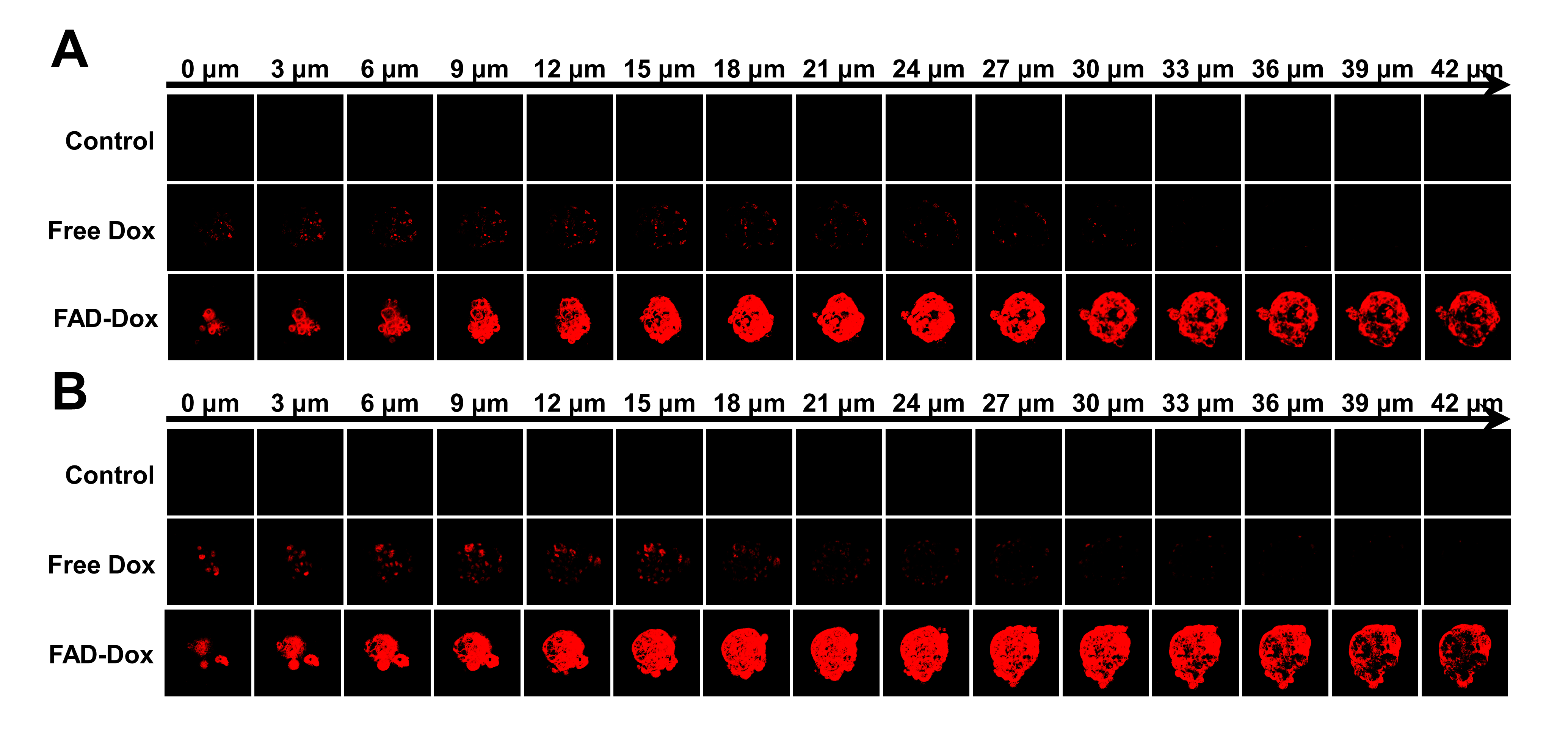


# References

[1] X. Liu, J. Zhou, T. Yu, C. Chen, Q. Cheng, K. Sengupta, Y. Huang, H. Li, C. Liu, Y. Wang, P. Posocco, M. Wang, Q. Cui, S. Giorgio, M. Fermeglia, F. Qu, S. Pricl, Y. Shi, Z. Liang, P. Rocchi, J. J. Rossi, L. Peng, Angew. Chem. Int. Ed. 2014, 53, 11822-11827.

[2] N.E. Olesen, P. Westh, R. Holm, Journal of Colloid and Interface Science, 2015, 453, 79-89.
